# An *NKX2-1*^*GFP*^* and TP63*^*tdTomato*^ dual fluorescent reporter for the investigation of human lung basal cell biology

**DOI:** 10.1038/s41598-021-83825-6

**Published:** 2021-02-25

**Authors:** Kim Jee Goh, Ee Kim Tan, Hao Lu, Sudipto Roy, N. Ray Dunn

**Affiliations:** 1 Institute of Medical Biology, Agency for Science Technology and Research (A∗STAR), 8A Biomedical Grove, #06-06 Immunos, Singapore, 138648 Singapore; 2 Skin Research Institute of Singapore, 11 Mandalay Road #17-01 Clinical Sciences Building, Singapore, 308232 Singapore; 3 Lee Kong Chian School of Medicine, Nanyang Technological University, Clinical Sciences Building, 11 Mandalay Road, Singapore, 308232 Singapore; 4 Institute of Molecular and Cell Biology, Proteos, 61 Biopolis Drive, Singapore, 138673 Singapore; 5 Department of Pediatrics, Yong Loo Lin School of Medicine, National University of Singapore, 1E Kent Ridge Road, Singapore, 119288 Singapore; 6 Department of Biological Sciences, National University of Singapore, 14 Science Drive 4, Singapore, 117543 Singapore

**Keywords:** Cell biology, Developmental biology, Stem cells

## Abstract

Basal cells are multipotent stem cells responsible for the repair and regeneration of all the epithelial cell types present in the proximal lung. In mice, the elusive origins of basal cells and their contribution to lung development were recently revealed by high-resolution, lineage tracing studies. It however remains unclear if human basal cells originate and participate in lung development in a similar fashion, particularly with mounting evidence for significant species-specific differences in this process. To address this outstanding question, in the last several years differentiation protocols incorporating human pluripotent stem cells (hPSC) have been developed to produce human basal cells in vitro with varying efficiencies. To facilitate this endeavour, we introduced *tdTomato* into the human *TP63* gene, whose expression specifically labels basal cells, in the background of a previously described hPSC line harbouring an *NKX2-1*^*GFP*^ reporter allele. The functionality and specificity of the *NKX2-1*^*GFP*^*;TP63*^*tdTomato*^ hPSC line was validated by directed differentiation into lung progenitors as well as more specialised lung epithelial subtypes using an organoid platform. This dual fluorescent reporter hPSC line will be useful for tracking, isolating and expanding basal cells from heterogenous differentiation cultures for further study.

## Introduction

Respiratory diseases are leading causes of death and disability in the world. About 65 million people suffer from chronic obstructive pulmonary disease (COPD) and 3 million die from it each year, making it the third leading cause of death worldwide^1^. In order to develop preventative and/or curative strategies against lung diseases, understanding lung development, regeneration and repair are key to dissecting disease mechanisms.

The lungs are made up of an extensive branching network of epithelial tubes. Air enters the nasal passages, flows down the trachea into increasingly smaller branches of bronchioles until it reaches the alveoli where gas exchange takes place. In addition to the epithelial cells that line the respiratory tract, the lungs are also home to various other cell types such as smooth muscle cells, fibroblasts, immune cells and neurons, all of which play important roles in lung homeostasis and function. With the constant interaction between the epithelial cells and the external environment, cell loss due to natural turnover, damage or disease is inevitable. The adult lung, although relatively quiescent compared to organs with high cellular turnover such as the intestine, possesses a remarkable ability to regenerate and repair itself following acute injury^2,3^. Rodent injury models, lineage tracing and transcriptome analyses have been useful in the identification and study of the cell types involved in this process^4–19^.

Basal cells are a population of multipotent stem cells with the ability to self-renew and regenerate all the epithelial subtypes present in the proximal region of the lung^20^. Named because of their location next to the basal lamina, basal cells are present throughout the trachea and terminal bronchioles in humans. Basal cells can be identified through expression of *Transformation-related protein 63* (*TP63*), *Cytokeratin 5* (*KRT5*), *Nerve Growth Factor Receptor* (*NGFR*) and *Integrin subunit Αlpha 6* (*ITGA6*)^21^. Expression of *TP63* appears to be crucial to basal cell identity as mice without functional *Trp63* lack basal cells and die at birth^22–24^. Lung development begins at embryonic day (E)9 in the mouse and around 4 weeks post-conception (pcw) in humans. Expression of *Nkx2-1* is crucial to this process as *Nkx2.1* mutant mice fail to develop lungs and humans with *NKX2-1* mutations develop congenital lung diseases^25–27^. These Nkx2-1 + cells in the anterior foregut form the lung buds that then undergo branching morphogenesis. Proximal–distal patterning occurs whereby acquisition of *Sox2* or *Sox9* expression directs differentiation of these early lung progenitors toward proximal or distal lineages respectively. Basal cells were thought to emerge later during lung development^28^ until a recent study by Yang et al. demonstrated using multiple *Cre* recombinase mouse driver lines, that Trp63 + basal cells appear shortly after the initiation of lung development at E9.5. These early basal cells, although capable of contributing to both proximal and distal epithelial cell lineages, become more lineage-restricted by E10.5^29^.

Tremendous progress has been made in understanding lung development with the aid of murine models. However, with increasing evidence of biologically significant differences between murine and human lungs^30,31^, it is important that this knowledge gap be filled in order to understand human-specific disease mechanisms. Nonetheless, even with the lack and/or sparse amount of human data, several groups have developed protocols largely based on mimicking in vivo mouse lung development to direct differentiation of human pluripotent stem cells (hPSCs), whether human embryonic stem cells (hESCs) or human induced pluripotent stem cells (hiPSCs), into lung epithelial cells^32–39^. These protocols generate lung progenitors with varying degrees of efficiency that can be further matured into a variety of lung epithelial cell subtypes. Initial studies were aimed at increasing the yield of NKX2-1 + lung progenitors. Interest is however mounting in directing the differentiation of hPSCs into specific lung lineages^36,37,39^. Despite these efforts, the origins and development of human lung basal cells remain unknown. Given the important role these cells play in lung homeostasis and repair, elucidating the molecular mechanisms of their development can potentially inform the development of protocols to direct hPSC differentiation into basal cells, which will be invaluable in applications such as disease modelling, regenerative medicine as well as for the understanding of normal human lung development. To this end, we have generated an *NKX2-1*^*GFP*^*;TP63*^*tdTomato*^ dual fluorescence reporter line that will facilitate the investigation of human lung basal cell biology.

## Results

### Generation of the ***NKX2-1***^***GFP***^***;TP63***^***tdTomato***^ dual fluorescence reporter line

**Figure 1:**
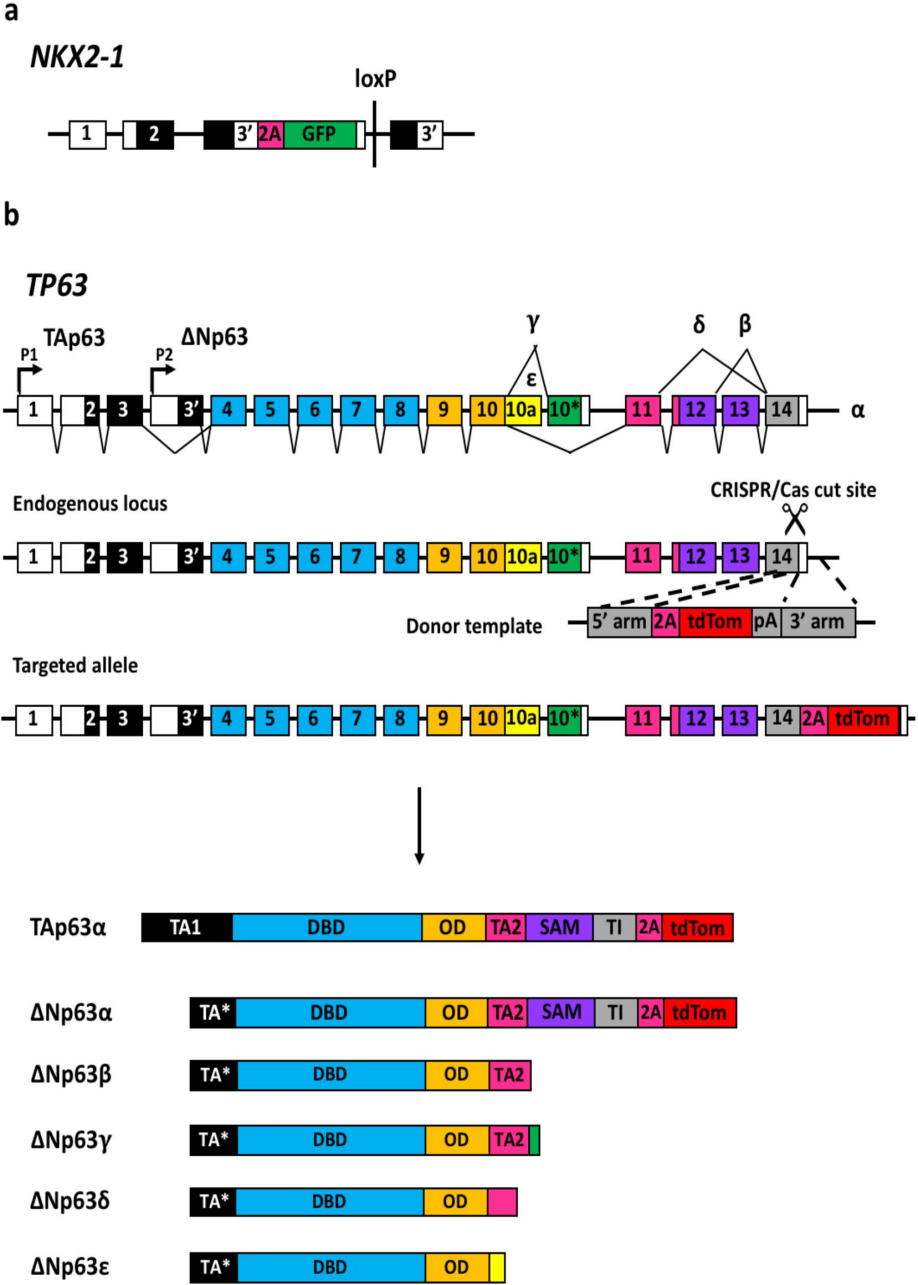
Schematic representation of the targeting strategy used to insert *tdTomato* into the endogenous *TP63* locus in the *NKX2-1*^*GFP*^ hiPSC reporter line^46^. (**a**) Schematic of targeted *NKX2-1* allele in BU3 *NKX2-1*^*GFP*^ hiPSC line from Hawkins et al.^46^. (**b**) Single guide RNAs were designed targeting the 3′ end of exon 14. Donor template consists of 800 bp homology arms flanking sequences encoding a P2A peptide (2A) and tdTomato. Expression of the α isoform of *TP63* (both *TAp63* and *ΔNp63*) is expected to lead to expression of *tdTomato*.

As *TP63* is important to basal cell identity and development, we introduced the *tdTomato* fluorescent reporter into the human *TP63* gene locus. The *TP63* gene is transcribed from two promoters, generating two isoforms with an N-terminal P53-homologous transactivation domain (TAp63) or without (ΔNp63). These isoforms undergo alternative splicing, yielding 10 different isoforms with 5 different C-termini designated α, β, γ, δ, and ε^40–43^. The α isoform is the longest isoform, incorporating exons 11 through 14 that encode the Sterile Alpha Motif (SAM) and a Post-Inhibitory Domain (PID). As *ΔNp63α* is the most highly expressed isoform in airway epithelial cells^44,45^, we chose to generate a *ΔNp63α* reporter allele in which *tdTomato* is inserted at the 3′ end of exon 14 in the previously described BU3 *NKX2-1*^*GFP*^ hiPSC line (Fig. 1A)^46^ that allows the specific isolation of lung basal cells (Fig. 1B)^47^.

Using the CHOPCHOP web tool (https://chopchop.cbu.uib.no), two single guide RNAs (sgRNAs) targeting the end of exon 14 were identified (Supplementary Fig. 1A). The T7EI assay was performed to assess cleavage efficiencies of the two sgRNAs. Cas9-mediated cleavage is predicted to result in a product of approximately 880 bp. Successful cleavage was observed with sgRNA 2 (Supplementary Fig. 1B). This sgRNA was then transfected into BU3 *NKX2-1*^*GFP*^ hiPSCs along with the donor template using Lipofectamine STEM. The donor template was constructed with 800 bp 5′ and 3′ homology arms flanking the *P2A-tdTomato* sequence. A total of 169 colonies were screened and only one potential homozygous clone was obtained (Supplementary Fig. 1C). This clone was then expanded, subcloned, genotyped and sequenced to confirm bi-allelic insertion of *P2A-tdTomato* (Supplementary Fig. 1D,E).

### Characterization of the *NKX2-1*^*GFP*^*; TP63*^*tdTomato*^ hiPSC line

*NKX2-1*^*GFP*^;*TP63*^*tdTomato*^ hiPSC colonies maintained typical ESC colony morphology (Supplementary Fig. 2A) and expressed the pluripotency marker OCT4 at levels similar to the BU3 parental line (Supplementary Fig. 2B,C). The modified hiPSC line displayed no gross chromosomal abnormalities (Supplementary Fig. 2D) and was also capable of differentiating into cells derived from all 3 germ layers (Supplementary Fig. 3), indicating that the insertion *of tdTomato* did not impact pluripotency.

**Figure 2:**
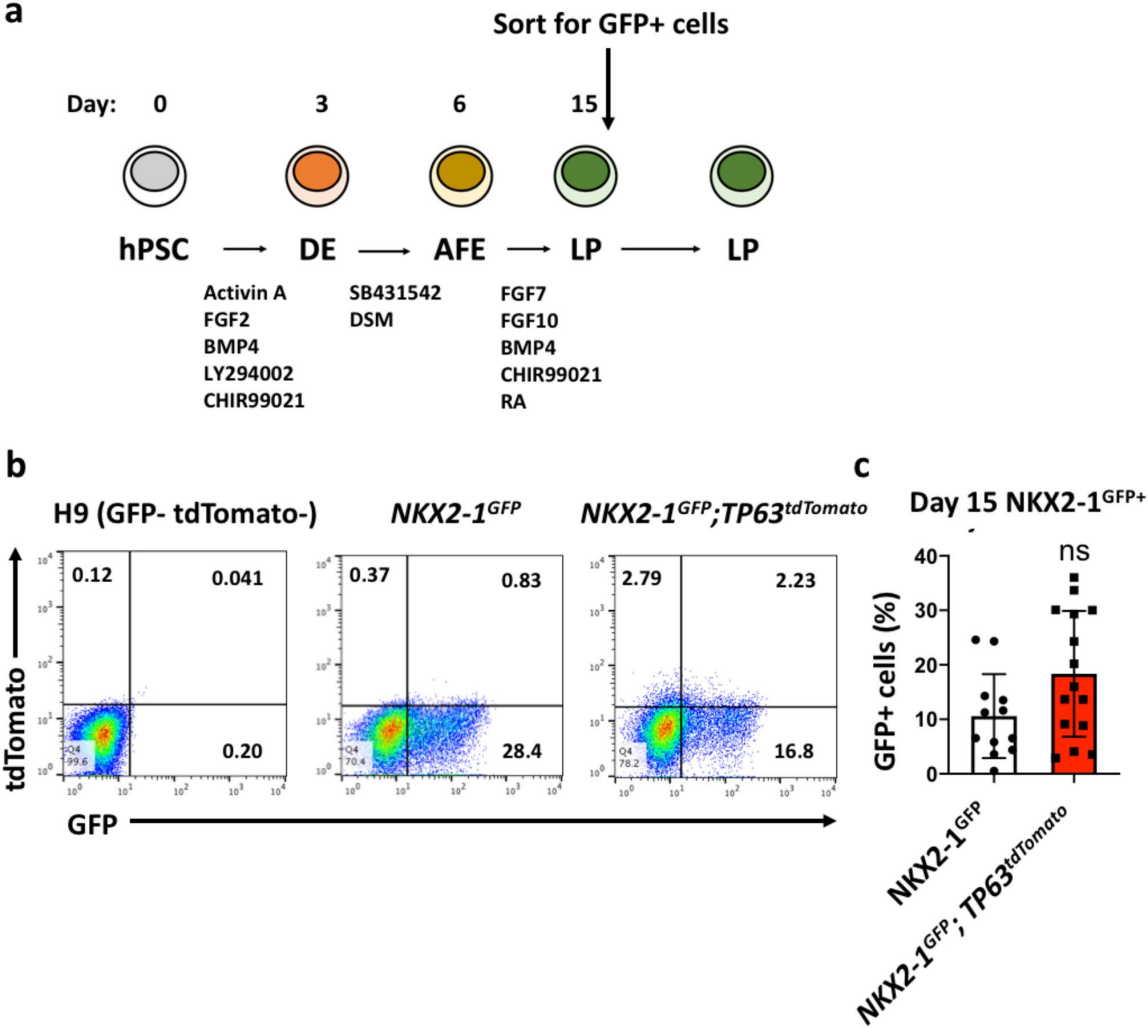
Assessment of tdTomato reporter functionality. (**a**) Schematic of lung differentiation protocol based on the work of McCauley et al*.* (2017)^38^. hPSC: human pluripotent stem cell; DE: definitive endoderm; AFE: anterior foregut endoderm; LP: lung progenitor. (**b**) Flow cytometry analysis of GFP + tdTomato + cells at Day 15 of the differentiation protocol. Representative dot plot shown. (**c**) Quantification of NKX2-1^GFP+^ cells at Day 15 as a measure of lung differentiation efficiency. Data are represented as means ± SD, n = 11 (*NKX2-1*^*GFP*^), n = 15 (*NKX2-1*^*GFP*^*; TP63*^*tdTomato*^). ns, not statistically significant. t test. Graph was made and statistical analysis was done using GraphPad Prism 8.0 (GraphPad Software Inc., San Diego, CA, USA) (www.graphpad.com).

To assess tdTomato reporter functionality, the parental and the *NKX2-1*^*GFP*^*;TP63*^*tdTomato*^ hiPSC lines were differentiated into early lung progenitors using a modified version of the protocols published by McCauley et al.^38^ (Fig. 2A). At day 15, NKX2-1^GFP+^ cells were observed in both lines, with a minority of cells tdTomato-positive (tdTomato^+^) (0.88 ± 1.01, n = 11) (Fig. 2B). Both lines differentiated into NKX2-1^GFP+^ lung progenitors at similar efficiencies (Fig. 2C). The insertion of *P2A-tdTomato* into the *TP63* locus therefore does not appear to impact lung differentiation efficiency.

**Figure 3:**
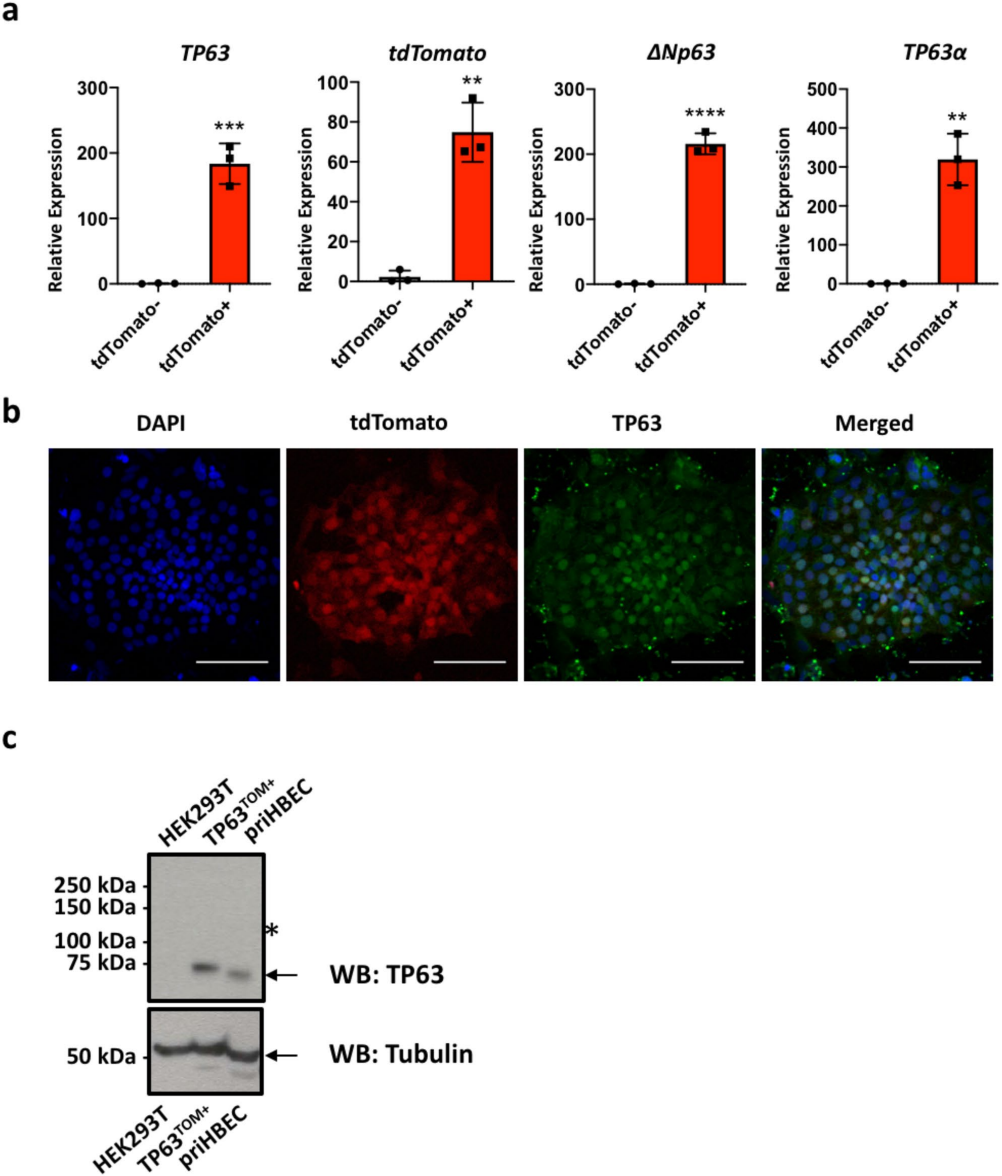
Assessment of tdTomato reporter specificity. (**a**) QPCR analysis of *TP63* (total), ∆*Np63*, *TP63α* and *tdTomato* expression in tdTomato- and tdTomato + fractions. n = 3. **p < 0.01, ***p < 0.001, ****p < 0.0001. t test. Graphs were made and statistical analyses were done using GraphPad Prism 8.0 (GraphPad Software Inc., San Diego, CA, USA) (www.graphpad.com). (**b**) Isolated tdTomato + cells plated onto Matrigel-coated plates and immunostained for TP63. Scale bar indicates 100 µm. (**c**) Western blot analysis of TP63 and Tubulin. 30 µg of whole cell lysates prepared from HEK293T cells overexpressing tdTomato (HEK293T), TP63TOM + cells, and primary human bronchial epithelial cells (priHBEC). Blots were probed with anti-TP63 antibody with Tubulin used as a loading control. Bands corresponding to TP63 and Tubulin are indicated by black arrows. The asterisk indicates where the uncleaved TP63-P2A-tdTomato protein of about 110 kDa is expected to be, if present. The full uncropped blots are presented in Supplementary Fig. 5.

To assess the specificity of the *TP63*^*tdTomato*^ reporter, the population of tdTomato^+^ cells that emerged on day 15 was collected via FACS sorting and mRNA harvested for analysis of *TP63* and *tdTomato* expression. Both *TP63* and *tdTomato* expression were found to be enriched in the tdTomato^+^ fraction (Fig. 3A). Immunostaining of tdTomato + cells with an anti-TP63 antibody showed co-localization of tdTomato and TP63 (Fig. 3B), demonstrating the faithfulness of the *TP63*^*tdTomato*^ reporter allele. Western blot was also performed to confirm enrichment of TP63 in the tdTomato + fraction (TP63^TOM+^). HEK293T cells overexpressing tdTomato were used as a negative control and primary human bronchial epithelial cells (priHBEC) were used as a positive control for TP63 (Fig. 3C). TP63 protein was detected only in the TP63TOM + and priHBEC samples. Uncleaved TP63-P2A-tdTomato protein (approximate expected size 110 kDa) was not detected in the TP63^TOM+^ samples, indicating efficient cleavage of the *P2A* sequence, consistent with previous reports on P2A being the most efficient self-cleaving peptide sequence^48,49^.

### Differentiation of the *NKX2-1*^*GFP*^*;TP63*^*tdTomato*^ hiPSC line into lung organoids

**Figure 4:**
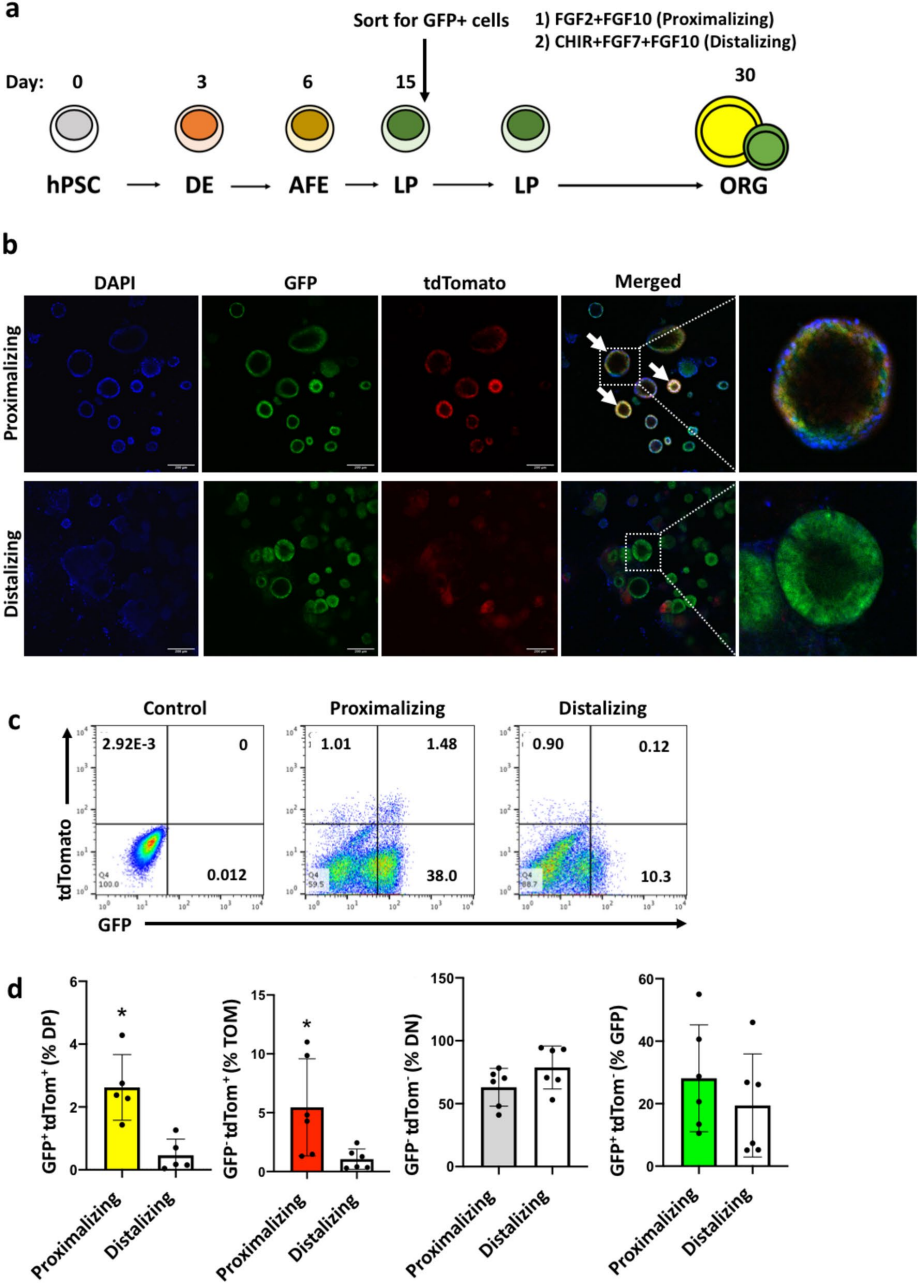
Differentiation of *NKX2-1*^*GFP*^*;TP63*^*tdTomato*^ hiPSCs into lung organoids. (**a**) Protocol for differentiating *NKX2-1*^*GFP*^*;TP63*^*tdTomato*^ hiPSCs into proximalized and distalized lung organoids based on McCauley et al.^38^ and Jacob et al.^39^. *hPSC* human pluripotent stem cell, *DE* definitive endoderm, *AFE* anterior foregut endoderm, *LP* lung progenitors, *ORG* lung organoid. (**b**) Representative images of proximalized and distalized lung organoids derived from the *NKX2-1*^*GFP*^*;TP63*^*tdTomato*^ hiPSC line. White arrows indicate lung organoids containing double positive cells. (**c**) Flow cytometry analysis of lung organoids cultured in proximalizing and distalizing conditions. Representative dot plots shown. (**d**) Quantification of the 4 populations in the lung organoids grown in the differentiation culture conditions. *DN* double negative (GFP- tdTOM-), *DP* double positive (GFP + tdTOM +), *tdTomato +* tdTomato-positive only (GFP- tdTOM +), *GFP* GFP-positive only (GFP + tdTomato-). Data are represented as means ± SD, n = 5. *p < 0.05. t test. Graphs were made and statistical analyses were done using GraphPad Prism 8.0 (GraphPad Software Inc., San Diego, CA, USA) (www.graphpad.com).

**Figure 5:**
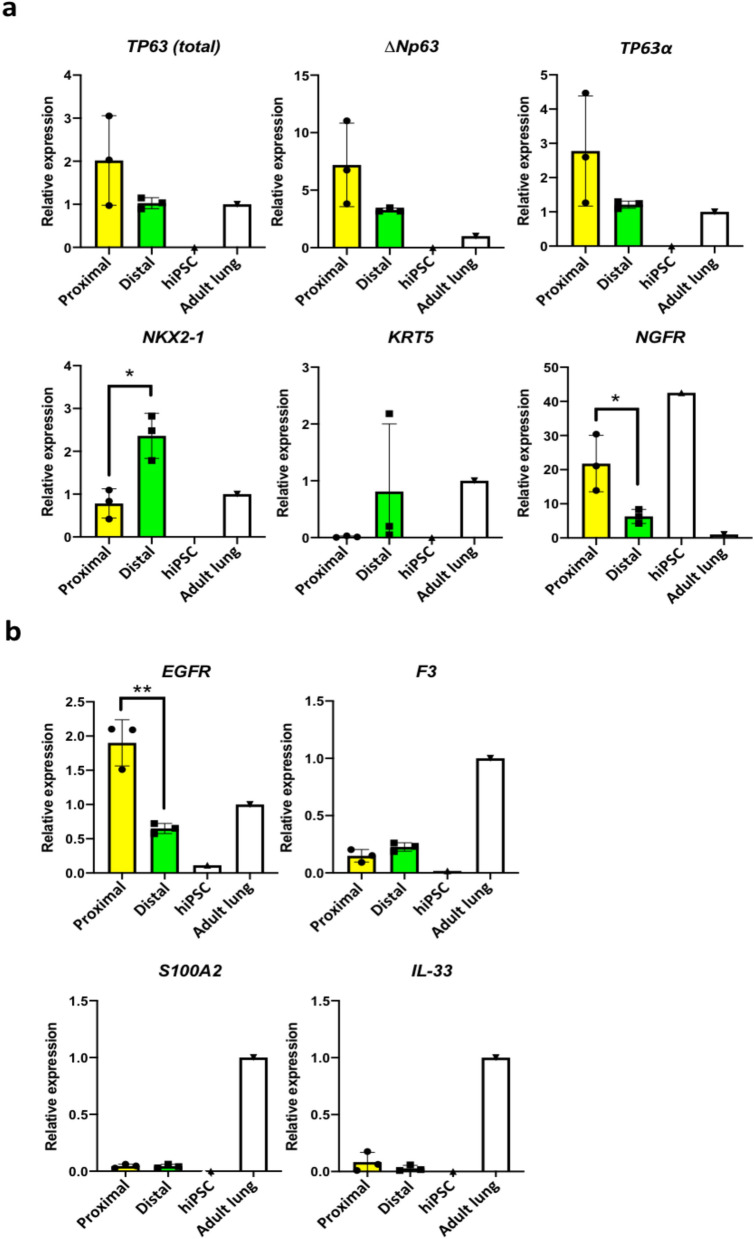
Gene expression analysis of *NKX2-1*^*GFP*^*;TP63*^*tdTomato*^ hiPSC-derived lung organoids. (**a**) QPCR analysis of lung marker *NKX2-1* and basal cell markers *TP63 (total), ΔNp63, TP63α*, *KRT5*, and *NGFR*. Expression levels were normalized to housekeeping gene, *ACTB.* n = 3. *p < 0.05. t test. (**b**) QPCR analysis of additional markers identified by Miller et al*. (*2020)^50^ to be associated with basal cells. Expression levels were normalized to *ACTB*. n = 3. **p < 0.01. All graphs were made and statistical analyses were done using GraphPad Prism 8.0 (GraphPad Software Inc., San Diego, CA, USA) (www.graphpad.com).

McCauley et al. and Jacob et al*.* have shown that modulation of WNT signalling in early lung progenitors can influence the specification and development of lung cell types^38,39^. Specifically, activation of WNT signalling induces distal lung fates while its absence promotes proximal lung fates. Indeed, BU3 *NKX2-1*^*GFP*^ hiPSCs can be differentiated into lung organoids expressing mainly proximal and distal lung markers in low-WNT and high-WNT conditions, respectively (Supplementary Fig. 4). We therefore hypothesized that low-WNT conditions should generate more NKX2-1^GFP+^;TP63^tdTomato+^ cells compared to high WNT conditions. NKX2-1^GFP+^ cells were thus isolated from differentiated *NKX2-1*^*GFP*^*;TP63*^*tdTomato*^ hiPSCs via FACS sorting, embedded in Matrigel drops and then grown as organoids for 2 weeks in proximalizing^38^ or distalizing^39^ culture medium (Fig. 4A). Organoids that formed in the proximalizing conditions were observed to contain more GFP^+^ tdTomato^+^ double positive (DP) cells compared to the distalizing conditions (Fig. 4B). Flow cytometry was performed on dissociated organoids to quantify the cells in the double negative, tdTomato^+^, GFP^+^ and DP fractions (Fig. 4C). Proximalizing conditions yielded higher numbers of DP as well as tdTomato^+^ cells compared to the distalizing conditions (Fig. 4D). Consistent with previous reports^38,39^, the CHIR99021-containing conditions promoted a more distal lung fate. QPCR analysis of organoids did not show significantly higher levels of *TP63* expression in the proximalized organoids (Fig. 5A). Interestingly, expression of *KRT5* was also found to be higher in the distalized conditions. The distal marker *NKX2-1* was expectedly enriched in distalized organoids, while the basal cell marker *NGFR* was found to be higher in the proximalized organoids. Miller et al*.* recently reported that the expression of *EGFR*, *IL-33*, *S100A2* and *F3* is enriched in early lung basal cells, and that EGFR and F3 can be used to purify these early lung basal cells from heterogeneous lung differentiation cultures^50^. Expression of these markers was also assessed via QPCR and expression of *EGFR* was significantly higher in the proximalized organoids (Fig. 5B).

### Characterization of NKX2-1^GFP^;TP63^tdTomato^ double positive (DP) cells

**Figure 6:**
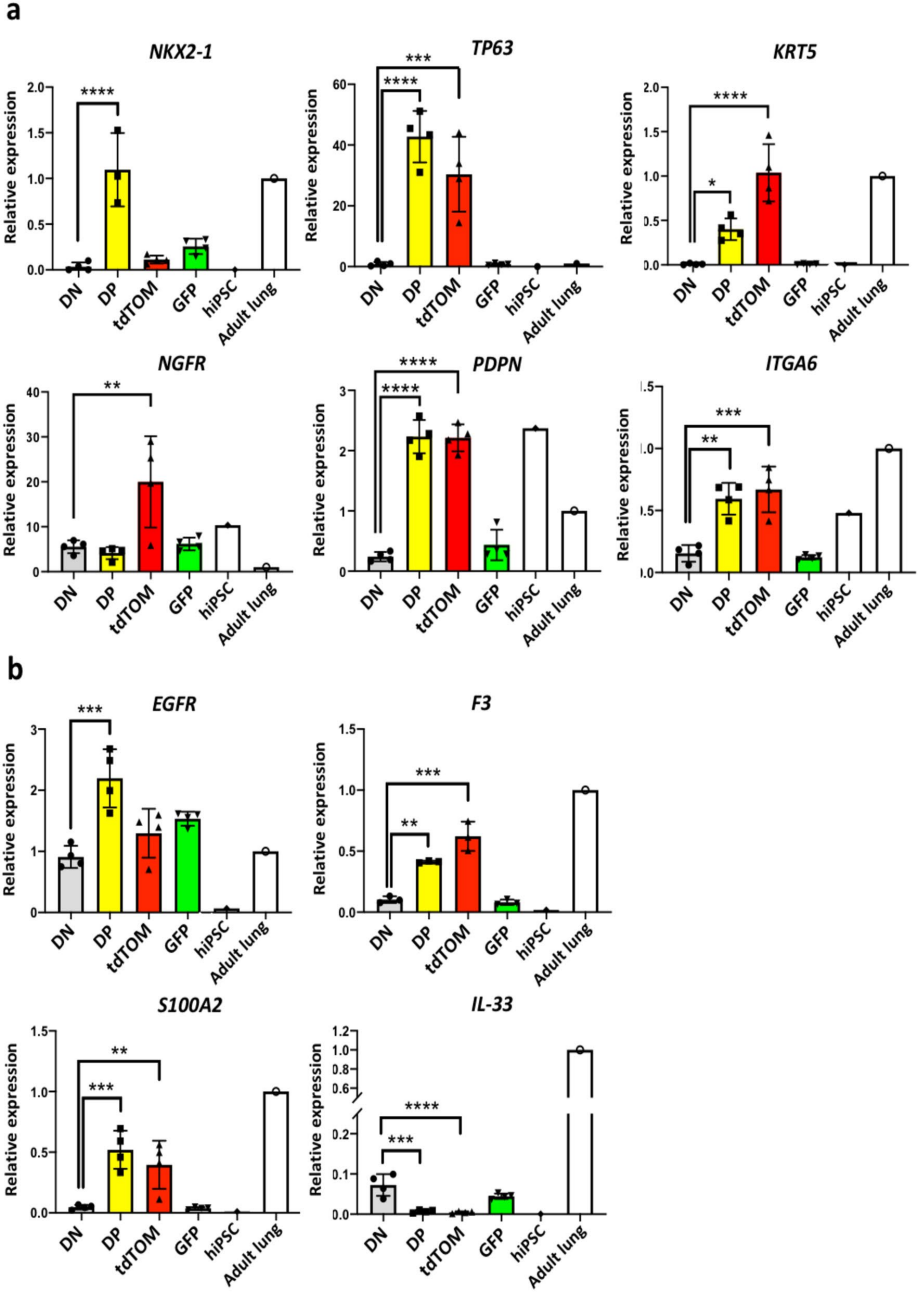
Analysis of basal cell marker expression in 4 cell fractions isolated from Day 30 proximalized lung organoids. (**a**) QPCR analysis of basal cell markers in DN, DP, tdTomato + and GFP + fractions isolated from Day 30 proximalized lung organoids. Expression levels were normalized to *ACTB*. *DN* double negative (GFP- tdTOM-), *DP* double positive (GFP + tdTOM +), *tdTomato + * tdTomato-positive only (GFP- tdTOM +), *GFP* GFP-positive only (GFP + tdTomato-), *hiPSC* undifferentiated BU3 *NKX2-1*^*GFP*^ hiPSCs. n = 4. **p < 0.01, ***p < 0.001, ****p < 0.0001. ANOVA followed by Tukey’s posthoc test. (**b**) QPCR analysis of other basal cell markers *EGFR*, *F3*, *S100A2* and *IL-33* in the DN, DP, tdTomato + and GFP + fractions. Expression levels were normalized to *ACTB*. n = 4. **p < 0.01, ***p < 0.001, ****p < 0.0001. ANOVA followed by Tukey’s post hoc test. All graphs were made and statistical analyses were done using GraphPad Prism 8.0 (GraphPad Software Inc., San Diego, CA, USA) (www.graphpad.com).

To confirm that the DP cells are specified to the basal cell lineage, the expression of other lung basal cell markers was assessed via QPCR. *TP63* was detected in both the DP and tdTomato^+^ fractions, confirming the specificity and functionality of the fluorescence reporter line (Fig. 6A). *NKX2-1* expression was present in both DP and GFP^+^ fractions and at low levels in the tdTomato^+^ fraction. *KRT5* was found to be expressed in the DP fraction but at higher levels in the tdTomato^+^ fraction. Expression of *NGFR* was not found to be increased in the DP cells compared to the double negative (DN) fraction. *NGFR* expression, however, was high in the tdTomato^+^ fraction. Expression levels of *EGFR*, *IL-33*, *S100A2* and *F3* were also assessed (Fig. 6B). Both DP and tdTomato^+^ cells expressed high levels of *EGFR*, *S100A2* and *F3* with DP cells expressing higher levels of *EGFR* and *S100A2*. *IL-33* was expressed at low levels in both DP and tdTomato^+^ cells.

**Figure 7:**
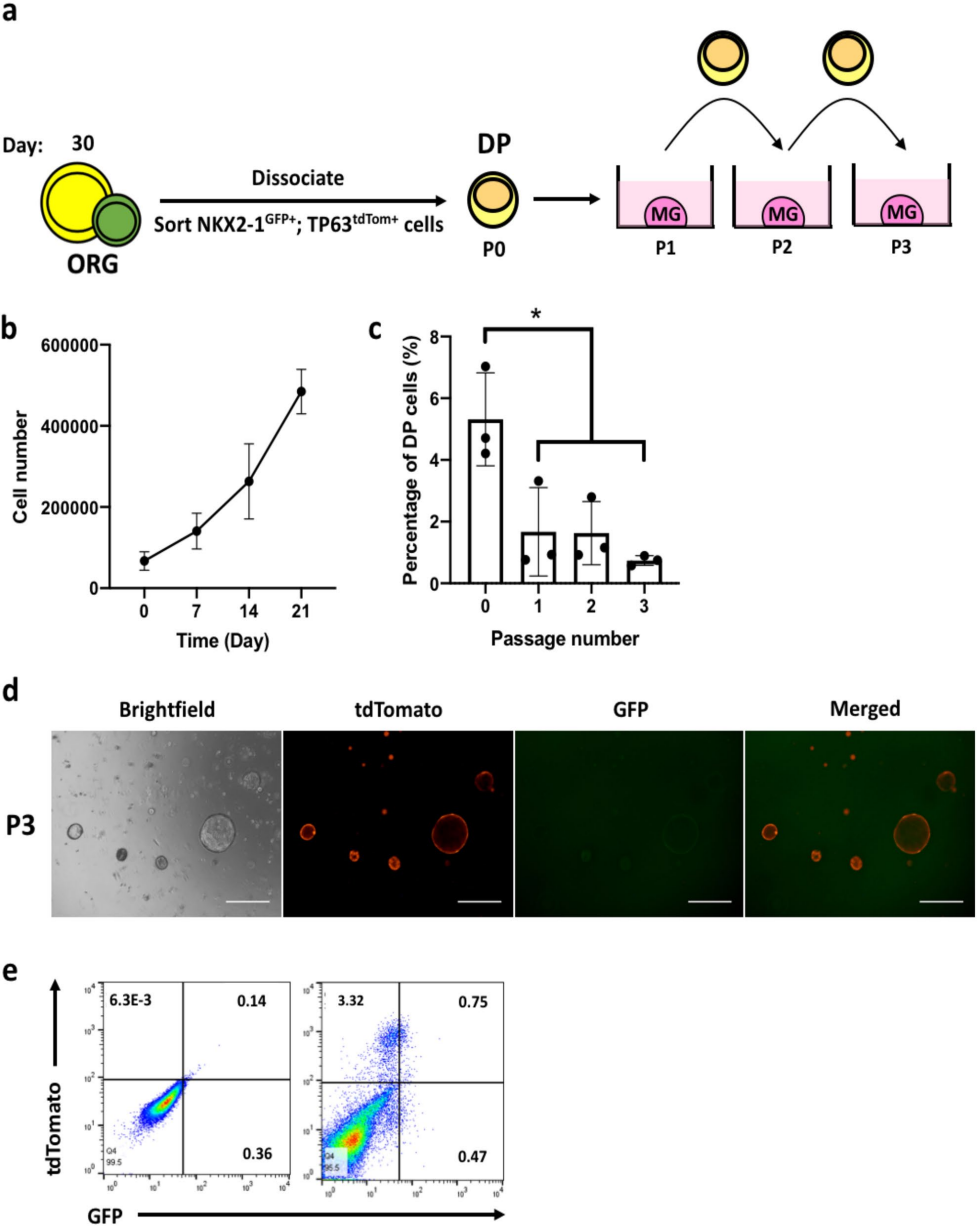
DP cells are proliferative and are capable of self-renewal. (**a**) Schematic of experimental setup. DP cells were serially passaged up to 3 passages. ORG: lung organoid; DP: double positive; MG: Matrigel. (**b**) Growth kinetics of DP cells at passage number 1 (P1). n = 3. (**c**) DP cells quantified at passage numbers P0, P1, P2 and P3 using flow cytometry. n = 3. (**b**,**c**) were made and statistical analyses were done using GraphPad Prism 8.0 (GraphPad Software Inc., San Diego, CA, USA) (www.graphpad.com). (**d**) Representative images of organoids derived from DP cells at P3. Scale bars indicate 500 µm. (**e**) Representative flow cytometry dot plot of dissociated organoids derived from DP P3 cells.

DP cells isolated from proximalized organoids (Passage number, P0) were passaged until P3 in proximalized culture conditions (Fig. 7A). Isolated DP (P0) cells were observed to be proliferative (Fig. 7B). However, we observed a gradual decrease in the proportion of DP cells with increasing passage number (Fig. 7C). DP cells at P3 were still capable of forming organoids in culture (Fig. 7D). While DP cells at P3 were still strongly TP63-positive and were capable of forming organoids in Matrigel, NKX2-1 expression was observed to be weak (Fig. 7D,E).

**Figure 8:**
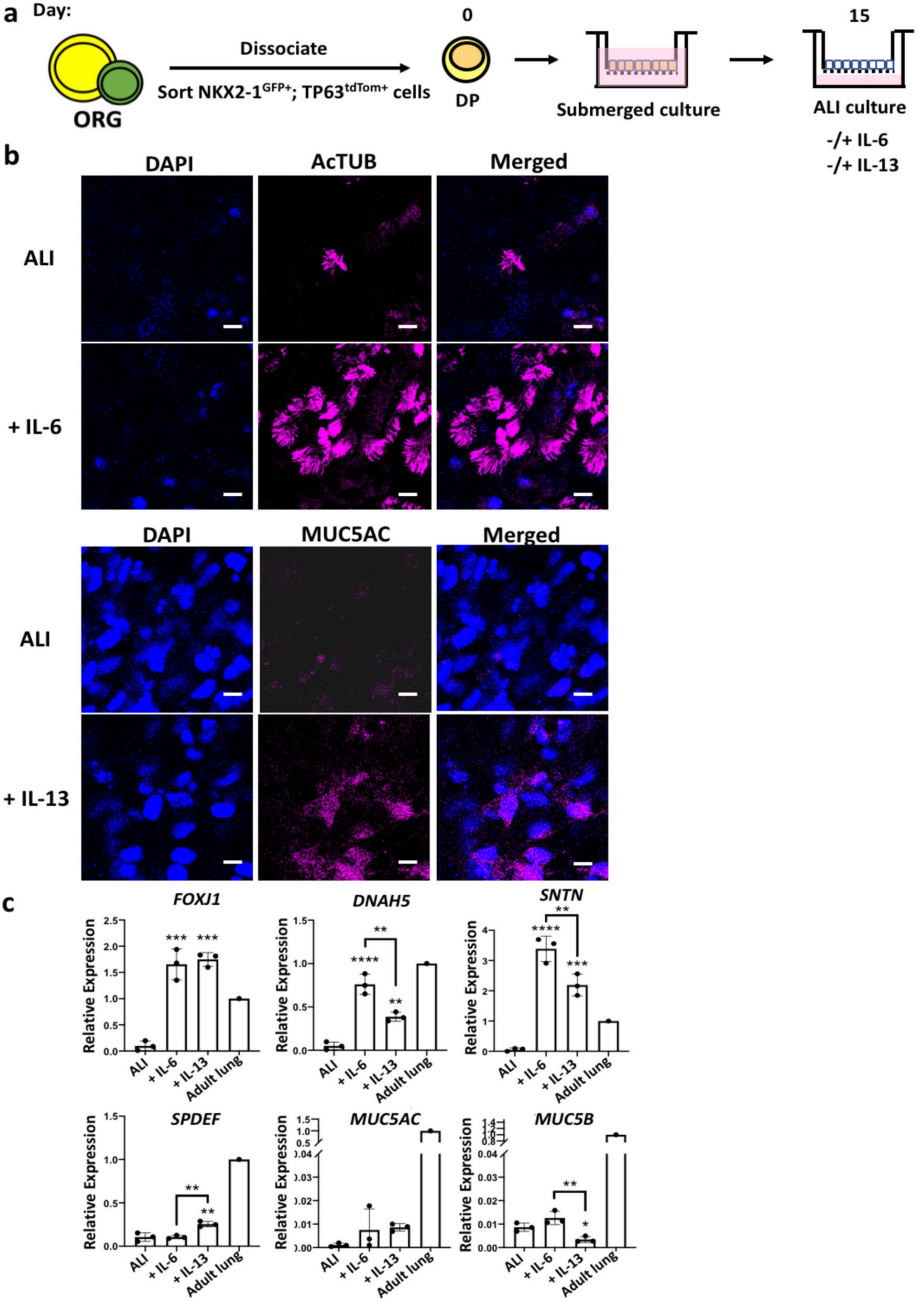
DP cells possess multilineage differentiation potential. (**a**) Schematic of differentiation protocol. Isolated DP cells were seeded onto transwells and then cultured at an air–liquid interface (ALI) upon reaching confluence in the absence or presence of 10 ng/ml IL-6 or 10 ng/ml IL-13. (**b**) Representative images of differentiation cultures immunostained for acetylated α-tubulin (AcTUB) and MUC5AC. Scale bars indicate 5 µm. n = 3. (**c**) QPCR analysis of multiciliated cell markers (*FOXJ1*, *DNAH5*, *SNTN*), secretory cell markers (*SPDEF*, *MUC5AC*, *MUC5B*). n = 3. Graphs were made and statistical analyses were done using GraphPad Prism 8.0 (GraphPad Software Inc., San Diego, CA, USA) (www.graphpad.com).

We next assessed whether DP cells are capable of multilineage differentiation like endogenous airway basal cells. DP cells were plated onto transwells, grown to confluence and then cultured at an air–liquid interface to induce terminal differentiation (Fig. 8A). Airway basal cells were also reported to respond to interleukin-6 (IL-6) and IL-13, factors purported to be able to increase differentiation of basal cells toward multiciliated and secretory cells, respectively^51–55^. Terminal differentiation was therefore performed in the presence of 10 ng/ml IL-6 or 10 ng/ml IL-13. DP cells were capable of differentiating into acetylated tubulin (AcTUB +) multiciliated cells and MUC5AC + secretory cells, and the addition of IL-6 or IL-13 altered the proportion of these cells in the ALI cultures (Fig. 8B). Ciliary movement could be observed in the ALI cultures (Supplementary video 1). Consistent with previous reports on the effects of IL-6 or IL-13 on differentiating airway epithelial cells, QPCR analysis for the expression of multiciliated cell markers (*FOXJ1*, *DNAH5* and *SNTN*) was increased in culture conditions containing IL-6 and IL-13 compared to the ALI only conditions (Fig. 8C)^51^. Although *FOXJ1* expression was not significantly different between IL-6- and IL-13-treated samples, levels of *DNAH5* and *SNTN* were observed to be lower in the IL-13 conditions compared to the IL-6 conditions, suggesting that in the presence of IL-6, basal cells preferentially differentiate into multiciliated cells over secretory cells. Moreover, DP cells differentiated in the presence of IL-13 express higher levels of *SPDEF* and *MUC5AC* and lower levels of *MUC5B* compared to the ALI only and IL-6-containing conditions. This is also in agreement with previous reports on the ability of IL-13 to skew the differentiation of basal cells toward the secretory cell lineage by increasing *MUC5AC* expression via STAT6/SPDEF^52–54,56^.

Taken together, our data show that the *TP63*^*tdTomato*^ faithfully reports endogenous *TP63* expression and can therefore be used to track and monitor basal cell development.

## Discussion

The airway epithelium is incredibly complex, consisting of many different specialized cell types. Lineage-tracing studies and the discovery of cell type-specific markers shed some light on the functions of these cell types in homeostasis and repair of the lung.

Here, we report the generation of an *NKX2-1*^*GFP*^;*TP63*^*tdTomato*^ hiPSC reporter line using CRISPR/Cas9 gene editing. Two other groups recently reported the generation of similar *NKX2-1*^*GFP*^*;TP63*^*tdTomato*^ hiPSC reporter lines^57,58^. Like Hawkins et al., we performed gene editing in the BU3 hiPSC line that harbours a bi-allelic integration of *GFP* into the endogenous *NKX2-1* locus^46^. In the second study, Drick et al. introduced the *tdTomato* reporter into the *TP63* locus in a previously characterized MHHi002-A-2 *NKX2-1*^*GFP*^ reporter hiPSC line^57,59^. However, the gene targeting strategies used by the aforementioned groups differ from ours in several ways. First, Drick et al. employed TALENs and inserted *tdTomato* into exon 10, while Hawkins et al. used CRISPR/Cas9 and inserted *tdTomato* into exon 4. Both groups selected exons present in all isoforms and splice variants of *TP63*^41,57,58^. We chose to introduce *tdTomato* at the 3′ end of exon 14 to monitor the expression of the *ΔNp63α* isoform, which is the most abundantly expressed isoform in airway epithelial cells. QPCR analyses showed that the *ΔNp63α* isoform is indeed the dominant isoform expressed in the hPSC-derived airway epithelial cultures and its expression pattern was similar to that of *tdTomato*. Insertion of the *tdTomato* reporter did not interfere with the maintenance of pluripotency and was also shown to be functional and specific to *ΔNp63α* expression. Second, the *tdTomato* reporter was integrated into only one allele in the reporter lines generated by both Drick et al*.* and Hawkins et al*.*, whereas our gene targeting approach resulted in homozygous integration of the *tdTomato* reporter in the *TP63* locus. Third, one additional, though likely subtle difference among the three gene-targeting strategies, is that Drick et al*.* and Hawkins et al*.* relied on the use of antibiotic selection cassettes while our targeting strategy did not. In the case of Hawkins et al*.* , the puromycin cassette was subsequently removed via Cre-loxP excision. Finally, modifications to the *TP63* locus in this study result in no loss of wild-type *TP63* expression, which is particularly important given the key role *TP63* plays in a multitude of cellular processes and that heterozygous mutations in *TP63* have been reported to result in developmental disorders and diseases in humans^60–71^.

The *NKX2-1*^*GFP*^*; TP63*^*tdTomato*^ dual fluorescent reporter hiPSC line was differentiated into proximalized and distalized lung organoids using published protocols to assess tdTomato reporter specificity and functionality. Proximalized lung organoids derived from the *NKX2-1*^*GFP*^*;TP63*^*tdTomato*^ hiPSC reporter line were observed to contain significantly higher numbers of *NKX2-1*^*GFP*+^*;TP63*^*tdTomato*+^ DP cells compared to the distalized lung organoids, quantified by flow cytometry. This is in line with previous reports by McCauley et al*.* and Jacob et al*.*^38,39^. However, QPCR analyses of these proximalized and distalized organoids did not indicate statistically significant higher levels of *TP63* (total), *ΔNp63* and *TP63α* in the proximalized compared to the distalized organoids. *KRT5* expression was observed to be higher in the distalized organoids although this is not statistically significant. This observation could be attributed to the NKX2-1^GFP-^;TP63^tdTomato+^ (TOM +) cells often present at the bottom of the wells underneath the organoids in the distalizing culture conditions. It is unclear if these cells have been derived from NKX2-1^GFP+^;TP63^tdTomato-^ (GFP +) or DP populations or simply non-lung cell types that were not eliminated during FACS sorting on day 15. It is possible that the TOM + population represents mature basal cells, but this needs to be confirmed via lineage tracing experiments. This underscores the importance of quantifying the number of DP present in the culture conditions via flow cytometry instead of QPCR.

We show that DP cells express basal cell markers (*KRT5, PDPN* and *ITGA6)* as well as markers recently identified by Miller et al*.* such as *EGFR*, *F3*, and *S100A2*^50^. Co-expression of *KRT5* and *NGFR* are thought to identify mature basal cells in both mice and humans^20,72^ and have been used as read-outs for basal cell maturity^73,74^. Interestingly, the DP population did not appear to express significantly higher levels of NGFR compared to the DN population. The low expression of *NGFR* in DP cells was also reported by Hawkins et al. who further showed that culturing DP cells in PneumaCULT Ex medium supplemented with A-83–01, Dorsomorphin and Y-27632 (BC medium), increased *NGFR* expression and induced basal cell maturity^58^. The TOM + cell population was observed to also express these markers at similar or higher levels (in particular, *NGFR* and *KRT5*) compared to the DP population. However, *KRT5* and *NGFR* are also expressed in basal cells of other epithelial tissues/organs such as the skin, cornea, esophagus, mammary gland and prostate^75–79^. It is therefore possible that these cells are non-lung cell types. The presence of the *NKX2-1*^*GFP*^ fluorescent reporter is particularly important in facilitating the specific isolation of airway basal cells.

Finally, we validated our dual fluorescent reporter hiPSC line by further characterizing the DP cell population. DP cells are proliferative but the proportion of DP cells was observed to decrease with increasing passage number. This could be due to the proximalizing culture conditions being sub-optimal for long term culture of airway basal cells. Similarly, primary airway basal cells have a short lifespan in culture, which can be extended through the use of various culture methods such as the small molecule inhibitors or feeder cells^80,81^. Indeed, Hawkins et al*.* showed that the hiPSC-derived basal cells can be maintained and expanded up to 10 passages when cultured in PneumaCULT-Ex medium supplemented with A-83–01, Dorsomorphin and Y-27632^58^. NKX2-1 expression was also observed to be decreased in these DP cells and their derived organoids. The downregulation of NKX2-1 expression could be due to the DP cells becoming more lineage-restricted with increasing time in culture. During lung development in the mouse, expression of NKX2-1 becomes increasingly confined to distal airway cells as branching morphogenesis occurs^82,83^. In addition, knockout mouse models indicate that Nkx2-1 is necessary for distal lung development but appears to be dispensable for proximal lung specification and morphogenesis^25,84^. Like primary airway basal cells, DP cells can also be terminally differentiated into multiciliated and secretory cells in ALI cultures. DP cells are also responsive to IL-6 and IL-13, which bias differentiation of basal cells toward multiciliated and secretory cells, respectively.

We anticipate that this dual fluorescent reporter hiPSC line will be very useful for the identification and isolation of lung basal cells for the study of basal cell development and differentiation. In addition, this line will also be a valuable tool in the development of culture conditions that enable the expansion and long-term maintenance of basal cells for a variety of applications^85^.

## Materials and methods

### Human pluripotent stem cell culture

BU3 *NKX2-1*^*GFP*^ hiPSCs were a kind gift from Professor Darrell Kotton, Boston University University School of Medicine, and were routinely cultured on Vitronectin-coated tissue culture dishes in Essential 8 (E8) medium (Life Technologies). All cells were maintained in an incubator with 5% CO_2_, at 37 °C.

### Construction of CRISPR sgRNA vectors and donor template

CRISPR sgRNAs targeting exon 14 of the human *TP63* gene were designed using CHOPCHOP (https://chopchop.cbu.uib.no/). Two sgRNAs were identified (*TP63* sgRNA 1 and sgRNA2), annealed and cloned into the pHF1-Cas9 plasmid. The donor template was constructed with the *P2A-tdTomato* cassette flanked by 800 bp homology arms amplified from genomic DNA extracted from the BU3 *NKX2-1*^*GFP*^ hiPSC line. Site-directed mutagenesis was performed to remove the PAM site on the donor template to prevent cleavage by Cas9. Primer sequences are found in Supplementary Table 1.

### Transfection

BU3 *NKX2-1*^*GFP*^ hiPSCs were plated onto vitronectin-coated tissue culture plates at 30% confluence one day before transfection. 2 µg of sgRNA and 7 µg donor template were diluted in OptiMEM, mixed at a 1:4 ratio with Lipofectamine STEM (Life Technologies) and then applied to the cells. E8 culture medium containing 5.25 µg/ml Blasticidin was supplied to the cells 24 h after transfection. Selection continued for two days, after which cells were fed fresh E8 medium daily until colonies were large enough for picking and genotyping.

### T7EI assay

BU3 *NKX2-1*^*GFP*^ hiPSCs transfected with 2 µg of empty vector or sgRNA using Lipofectamine STEM were harvested 7 days post-transfection. Genomic DNA was extracted using the QiAmp DNA mini kit (Qiagen). Gene-specific primers were used to amplify genomic regions flanking the sgRNA target sites via PCR. PCR products were purified using the QIAQuick PCR purification kit, denatured and allowed to reanneal in NEB buffer 2. The PCR products were then treated with or without T7 endonucleause I (NEB) at 37 °C for 1 h before they were resolved on a 2% agarose gel and visualized using an UVIpro Gel documentation system (UVItec).

### Genotyping

Genomic DNA was extracted from picked colonies in a 20 µl reaction volume containing 1X detergent (0.05% IGEPAL CA630, 0.05% Tween-20), proteinase K and 1X TE buffer, incubated at 55 °C for 1 h followed by a 5 min incubation at 95 °C. 0.5 to 1 µl of the reaction was then used in a 10 µl PCR reaction containing 1X Primestar Max Mastermix (Takara) and 1 µl 10 µM forward and reverse primers. The thermal cycling conditions used were as follows: 10 s at 98 °C, followed by 35 cycles of 10 s at 98 °C, 5 s at 55 °C and 1 min at 72 °C.

The primers used for genotyping were designed to target outside the 5′ and 3′ homology arms. A positive clone (with insertion of P2A-tdTomato) would yield a PCR product of 3.1 kb while a negative clone (wild-type) would yield a PCR product of 1.5 kb. Positive clones identified were sent for Sanger sequencing to confirm presence of P2A-tdTomato without any unwanted mutations. Primer sequences can be found in Supplementary Table 1.

### Clonal expansion

Positive BU3 *NKX2-1*^*GFP*^ hiPSC clones with P2A-tdTomato correctly integrated into the locus were dissociated into single cells using Accutase and seeded at very low densities onto vitronectin-coated plates in E8 medium supplemented CloneR (Stem Cell Technologies) per manufacturer’s instructions. E8 medium was refreshed daily until colonies emerged and were large enough for picking and genotyping. Homozygous clones were expanded in culture and frozen stocks were made using Cryostor (Stem Cell Technologies).

### Trilineage differentiation

hiPSCs were washed with DPBS once and then incubated with Accutase for 5 min at 37 °C. A P1000 pipette was then used to resuspend the cells and break up large cell clumps. Accutase was diluted out using DMEM F12 Advanced. The cell suspension was centrifuged at 1200 rpm for 5 min to collect cells into a pellet. The pellet was then resuspended in MEF medium (15% FBS, 1% Glutamax in DMEM F12 Advanced) and transferred into low adhesion tissue culture dishes to form embryoid bodies. The medium was changed every other day for 7 days after which the embryoid bodies were collected and allowed to attach onto 0.1% gelatin-coated tissue culture dishes. Cellular outgrowths were fed every other day for a further 7 days before they were fixed with 4% PFA for analysis of differentiation markers via immunofluorescence.

### Karyotype analysis

*NKX2-1*^*GFP*^*;TP63*^*tdTomato*^ hiPSC subclone 31.1 (Passage number P35 + 8, 8 passages post-subcloning) was grown to 70–80% confluency on a vitronectin-coated dish in E8 medium before cells were sent to the KK Women’s and Children’s Hospital (Singapore) Cytogenetics department for karyotype analysis.

### Flow cytometry

Cells were washed once with DPBS and then incubated with TrypLE express for 5 min at 37 °C. TrypLE express was then diluted out with DMEM F12 Advanced. Cells were collected into a pellet by centrifugation at 1200 rpm for 5 min at room temperature. Cell pellet was resuspended and fixed in 4% PFA and left to incubate for 20 min at room temperature with agitation every 5 min to prevent clumping. PFA was then removed and DPBS was added to wash the cells of residual PFA. The cell pellet was then collected via centrifugation at 1200 rpm for 5 min and then resuspended in 200 µl FACS buffer. 8 µl of isotype control antibodies or OCT4 antibodies were added and left to incubate in the dark for 1 h at room temperature. Excess and unbound antibodies were then removed and washed off by rinsing the cells with FACS buffer three times. Cells were then resuspended in 300 µl FACS buffer and passed through a 40 µm cell strainer to remove any cell clumps prior to analysis on the BD FACS Calibur. For the OCT4 staining experiment, the flow cytometry gates were set using Day 15 differentiated cells as a negative control. FlowJo was used to analyse all flow cytometry data. Antibodies used for FACS analyses can be found in Supplementary Table.

### Immunofluorescence

Cells were washed with DPBS and then fixed in 4% PFA for 20 min at room temperature. The PFA was removed and cells were washed with DPBS before they were blocked and permeabilized with blocking buffer (10% donkey serum, 0.1% Triton X-100 in DPBS) for 1 h at room temperature. Cells were then incubated with primary antibodies diluted in staining buffer (1% donkey serum, 0.1% Triton X-100 in DPBS) overnight at 4 °C. Excess and unbound primary antibodies were removed and the cells were washed 3 times with DPBS before incubated with fluorescence-conjugated secondary antibodies diluted in staining buffer for 1 h in the dark at room temperature. Cells were washed 3 times with DPBS to remove excess and unbound antibodies. NucBlue Fixed Cell ReadyProbes Reagent (DAPI, ThermoFisher Scientific) diluted in DPBS at 1 drop/ml was then added to each well to enable visualization of nuclei. Cells were then imaged using the Leica FV-3000 and ImageJ was used for image processing and analysis. The list of primary and secondary antibodies used can be found in the Supplementary Table 2.

To image organoids, media was removed from the wells and organoids embedded in Matrigel were carefully washed with DPBS once. The organoids were then fixed with 4% PFA for 20 min at room temperature. The PFA was then removed and the organoids were washed with DPBS once. 1 ml DPBS was then added to each well. 1 drop of DAPI solution was added to each well. Organoids were imaged using the Leica FV-3000 and images were analysed and processed using ImageJ.

### RNA extraction, cDNA synthesis and quantitative real-time PCR (QPCR)

Total RNA was extracted from cells using the Qiagen RNeasy kit per manufacturer’s instructions. 500 ng of purified RNA was converted into cDNA using the High-Capacity cDNA Reverse Transcription Kit (Applied Biosystems). QPCR was performed using the QuantStudio 7 Flex Real-Time PCR System. Samples were run in duplicates and normalized to β actin expression. Primer sequences can be found in Supplementary Table 3. Adult lung mRNA used as a positive control for QPCR experiments was obtained from Biochain (Total RNA – Human Adult Normal Tissue: Lung lower left lobe; Cat #R1234152-50; Lot #B6050780).

### Protein extraction and western blot

Whole cell lysates were prepared by lysing cells in Pierce RIPA buffer (ThermoFisher Scientific) supplemented with complete protease inhibitor cocktail (Calbiochem). 30 µg protein was resolved on a 7.5% SDS PAGE gel and then transferred onto PVDF membranes via semi-dry transfer method (Bio-rad). Membranes were blocked in 5% skim milk in Tris-buffered saline (TBST: 0.05 M Tris, 0.138 M NaCl, 0.0027 M KCl, pH 8.0) with 0.1% Tween-20 (Sigma-Aldrich) for an hour at room temperature. Membranes were then incubated with primary antibodies at 4 °C overnight. Membranes were then washed 3 times in TBST and then incubated with HRP-linked secondary antibodies for 1 h at RT. Membranes were washed 3 times with TBST before proteins were visualized using the SuperSignal West Femto Maximum Sensitivity Substrate (ThermoFisher Scientific) and Amersham Hyperfilm ECL (GE Healthcare). Primary and secondary antibodies can be found in Supplementary Table 2. Primary human bronchial epithelial cells used as a positive control for TP63 was obtained from Promocell (C-12640).

### Differentiation of hiPSCs into lung progenitors

hiPSCs were washed with DPBS and then incubated with Accutase for 5 min at 37 °C. A P1000 pipette was used to resuspend and break up clumps into single cells. Acctuase was diluted out using DMEM F12 Advanced and the cells were collected into a pellet by centrifuging at 1200 rpm for 5 min. The cell pellet was resuspended in E8 medium supplemented with 10 µM Y-27632. 400,000 cells were seeded into each 12-well and left in the incubator to attach overnight. Fresh E8 media was added to the cells the next day (Day -1, D-1). At D0, hiPSCs were differentiated into definitive endoderm (DE) cells using a protocol previously published by Vallier et al.^86^. To differentiate the DE cells into anterior foregut and subsequently lung progenitors, the protocol published by McCauley et al*.* was used^38^. Briefly, on D0, hiPSCs were treated 100 ng/ml Activin A, 80 ng/ml FGF2, 10 µM LY294002, 3 µM CHIR99021, 10 ng/ml BMP4 in CDM-PVA medium. On D1, hiPSCs were treated with 100 ng/ml Activin A, 80 ng/ml FGF2, 10 µM LY294002 and 10 ng/ml BMP4 in CDM-PVA medium. On D2, hiPSCs were treated with 100 ng/ml Activin A, 80 ng/ml FGF2, 1X B27, 1X NEAA in RPMI medium. To drive DE cells towards anterior foregut formation, cells were treated with 10 µM SB431542 and 2 µM Dorsomorphin (DSM) in basal medium (1X B27, 1X N2, 1X Glutamax, 1 mM HEPES in DMEM F12 Advanced) for 3 days. Finally, to differentiate AFE cells into lung progenitors, cells were treated with 3 µM CHIR, 10 ng/ml BMP4, 10 ng/ml FGF7, 10 ng/ml FGF10 and 50 nM RA in basal medium for 9 days.

### Lung organoid culture

Day 15-sorted GFP + cells were resuspended in basal medium containing 250 ng/ml FGF2, 100 ng/ml FGF10, 10 ng/ml FGF7, 50 nM Dexamethasone (Sigma-Aldrich), 0.1 mM 8-Bromoadenosine 30,50-cyclic monophosphate sodium salt (cAMP, Sigma-Aldrich) and 0.1 mM 3-Isobutyl-1-methylxanthine (IBMX) (Sigma-Aldrich), with or without the addition of 3 µM CHIR99021. Undiluted growth factor-reduced Matrigel (Corning) was added to the cell suspension at a 1:1 ratio after which 40 µl of this cell suspension was added to the middle of each 24-well plate to create a Matrigel drop. The plates were then returned to the incubator to allow the Matrigel drops to solidify for at least 30 min before medium is overlaid over the drops. Y-27632 was included in the medium for the first 24 h to improve cell survival. Medium was refreshed every other day for 2 weeks (Day 30) prior to analysis by flow cytometry.

### Fluorescence-activated cell sorting (FACS)

For sorting at Day 15, differentiated hiPSCs were washed once with DPBS and then incubated with TryPLE express for 15 min at 37 °C. A P1000 pipette was used to resuspend the cells to help break down large clumps into single cells. TrypLE express was diluted out with DMEM Advanced F12 and removed by centrifugation at 1200 rpm for 5 min. The cell pellet was resuspended in FACS buffer supplemented with 2% pen/strep and 10 µM Y-27632, passed through a 40 µm cell strainer to remove cell clumps and then sent to the Singapore Immunology Network (SIgN) Flow cytometry facility for sorting. H9 cells that are GFP- and tdTomato- were used as a negative control and used to set the gating parameters.

For sorting at Day 30, lung organoids embedded in Matrigel were washed with DPBS once. Organoids were then incubated with TrypLE express for 30 min at 37 °C. A P1000 pipette was used to resuspend and break the organoids down into single cells. DMEM F12 advanced was used to dilute out TrypLE express and then cells were collected into a pellet by centrifugation at 1200 rpm for 5 min. The cells were resuspended in FACS buffer supplemented with 2% pen/strep and 10 µM Y-27632, passed through a 40 µm cell strainer and then sent to the Singapore Immunology Network (SIgN) Flow cytometry facility for sorting.

### Terminal differentiation of DP cells

DP cells were seeded onto Matrigel-coated Transwell inserts. When cells reached 100% confluence, media was removed from both chambers and PneumaCULT ALI medium (Stem Cell Technologies) with or without 10 ng/ml IL-6 or 10 ng/ml IL-13 was added only to the bottom chamber. Medium was refreshed every other day for 2 weeks before cells were harvested for mRNA or fixed in 4% PFA for immunostaining.

### Statistical analysis

All graphs were made and statistical analyses were performed using GraphPad Prism 8.0 (GraphPad Software Inc., San Diego, CA). All error bars indicate mean ± standard deviation (SD). All experiments were performed with at least 3 biological replicates. The two-tailed Student’s T test was used to test for statistical significance where only a single parameter was compared between two groups. Where comparisons across > 2 groups had to be made, a one-way Analysis of Variance (ANOVA) was done followed by Tukey’s multiple comparisons analysis. A p value of < 0.05 was considered statistically significant.

## Supplementary Information

Supplementary Video 1.

Supplementary Information 1.
