# Supplementary material for: An NKX2-1GFP and TP63tdTomato dual fluorescent reporter for the investigation of human lung basal cell biology
Source: Sci Rep. 2021 Feb 25;11:4712. doi: 10.1038/s41598-021-83825-6 (PMC7907081; doi:10.1038/s41598-021-83825-6)
Supplement: Supplementary file 2 — Supplementary Information 1. [file 41598_2021_83825_MOESM2_ESM.pdf]

An *NKX2-1*^{GFP} *and TP63*^{tdTomato} dual fluorescent reporter for the investigation of human lung basal cell biology

Kim Jee Goh[1,2], Ee Kim Tan[1,3], Hao Lu[4], Sudipto Roy[4,5,6] and N. Ray Dunn[1,2,3]

[1] Institute of Medical Biology, Agency for Science Technology and Research (A∗STAR), 8A Biomedical Grove, #06-06 Immunos, Singapore 138648
[2] Skin Research Institute of Singapore, 11 Mandalay Road #17-01 Clinical Sciences Building, Singapore 308232
[3] Lee Kong Chian School of Medicine, Nanyang Technological University, Clinical Sciences Building, 11 Mandalay Road, Singapore 308232.
[4] Institute of Molecular and Cell Biology, Proteos, 61 Biopolis Drive, Singapore 138673
[5] Department of Pediatrics, Yong Loo Lin School of Medicine, National University of Singapore, 1E Kent Ridge Road, Singapore 119288
[6] Department of Biological Sciences, National University of Singapore, 14 Science Drive 4, Singapore 117543

*corresponding author: ray.dunn@ntu.edu.sg

Supplementary information

Supplementary figure 1

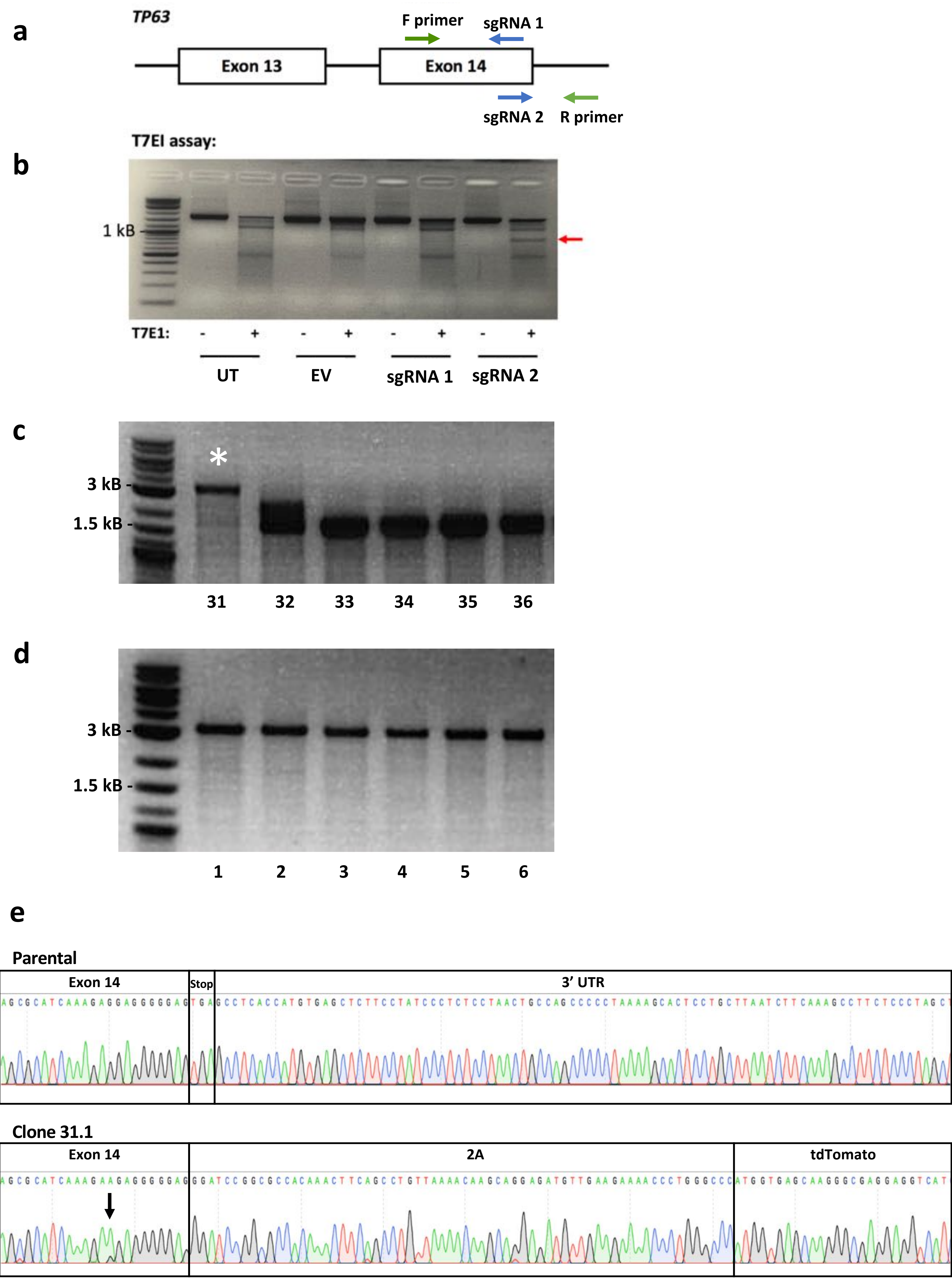

Supplementary figure 1. Generation of *NKX2-1^{GFP};TP63^{tdTomato}* hiPSC reporter line.

(a) Schematic showing exons 13 and 14 of TP63 gene and the targeting sites of sgRNAs 1 and 2. F and R primers used for genotyping clones target exon 14 and the 3’ UTR respectively.

(b) Gel image of T7EI assay. Red arrow indicates one of the expected band sizes (880 bp). Wild type band size is 1695 bp. UT: untransfected. EV: empty vector. Full gel image can be found at Supplementary Figure 6A.

(c) Gel image of 6 clones. Asterisk indicates the positive clone (Clone 31) with successful integration of tdTomato into the P63 locus. Full gel image can be found at Supplementary Figure 6B.

(d) Gel image of 6 subclones of Clone 31 showing that all subclones are homozygous for the tdTomato insertion. Subclone 31.1 was selected for further study. Full gel image can be found at Supplementary Figure 6C.

(e) Sanger sequencing chromatograms of parental line and Clone 31.1. The stop codon has been replaced by P2A sequence and tdTomato in Clone 31.1.

Supplementary figure 2

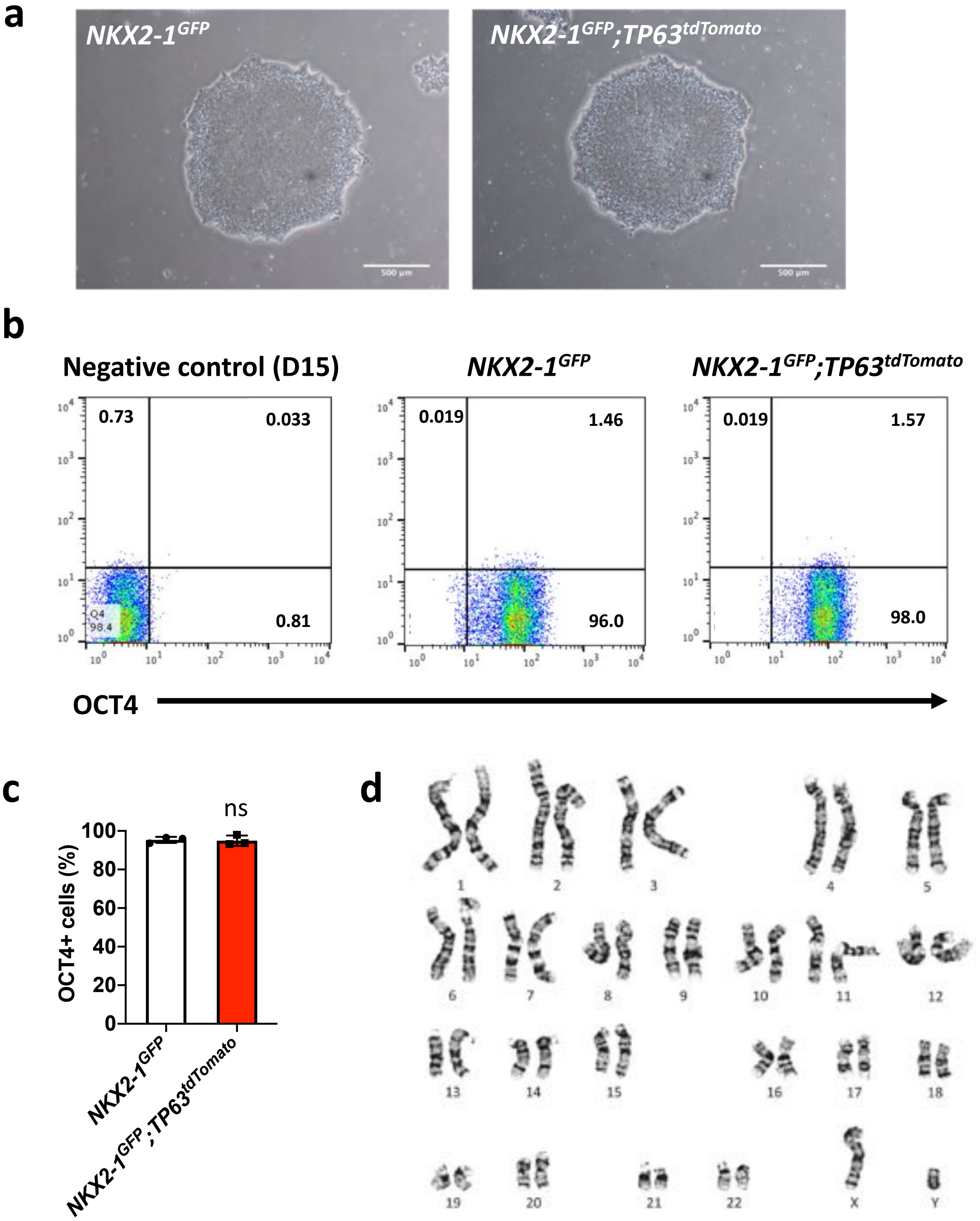

Supplementary figure 2. Characterization of *$NKX2\text{-}1^{GFP}$;$TP63^{tdTomato}$ iPSC* line.

(a) Brightfield images of parental and *$NKX2\text{-}1^{GFP}$;$TP63^{tdTomato}$* iPSC line.

(b) Flow analysis of OCT4+ cells in parental and *$NKX2\text{-}1^{GFP}$;$TP63^{tdTomato}$* iPSC line. Representative dot plot shown.

(c) Quantification of OCT4+ cells in parental and *$NKX2\text{-}1^{GFP}$;$TP63^{tdTomato}$* iPSC lines. Data represented as means ± SD, n=3. ns, not statistically significant. t test. Graph was made and statistical analysis was done using GraphPad Prism 8.0 (GraphPad Software Inc., San Diego, CA, USA) (www.graphpad.com).

(d) Karyotype of *$NKX2\text{-}1^{GFP}$;$TP63^{tdTomato}$* hiPSC subclone 31.1. Passage number P35+8 (8 passages post-subcloning).

Supplementary figure 3

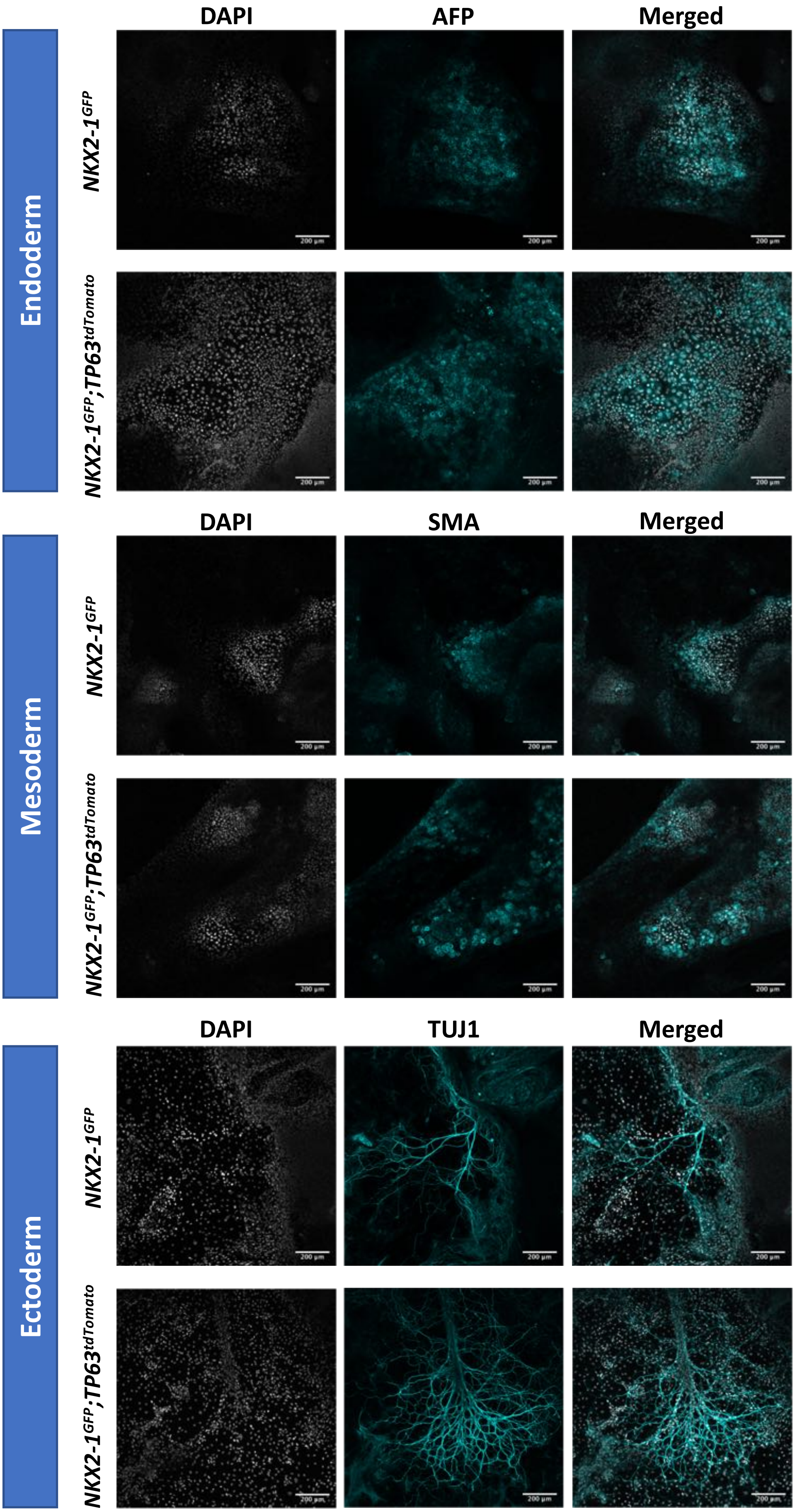

Supplementary figure 3. Trilineage differentiation of *NKX2-1^{GFP};TP63^{tdTomato}* iPSC.
Differentiation of *NKX2-1^{GFP};TP63^{tdTomato}* iPSC line into cells derived from the 3 germ layers and immunostained for AFP, SMA and TUJ1, markers of the endodermal, mesodermal and ectodermal lineages respectively.

Supplementary figure 4

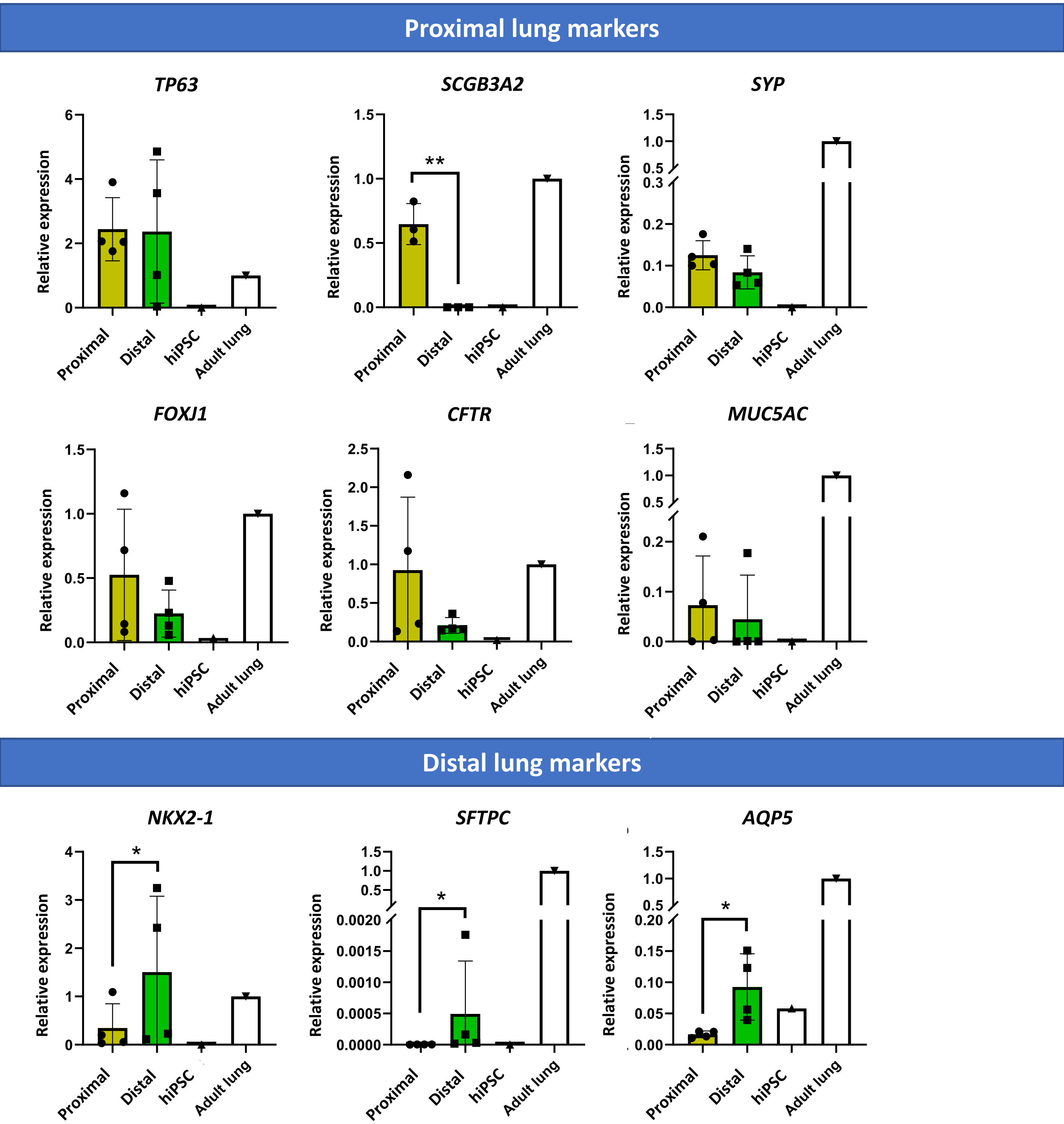

Supplementary Figure 4 – QPCR analysis of proximalized and distalized lung organoids derived from BU3 *NKX2-1* hiPSC line.

Day 30 proximalized or distalized organoids were assessed for expression of proximal lung markers *TP63*, *SCGB3A2*, *SYP*, *FOXJ1*, *CFTR* and *MUC5AC*, and distal lung markers *NKX2-1*, *SFTPC* and *AQP5*. Expression levels were normalized to housekeeping gene *ACTB.* hiPSC: Undifferentiated BU3 NKX2-1GFP hiPSCs. *$p<0.05$, **$p<0.01$. t test. All graphs were made and statistical analyses were done using GraphPad Prism 8.0 (GraphPad Software Inc., San Diego, CA, USA) (www.graphpad.com).

Supplementary figure 5

a

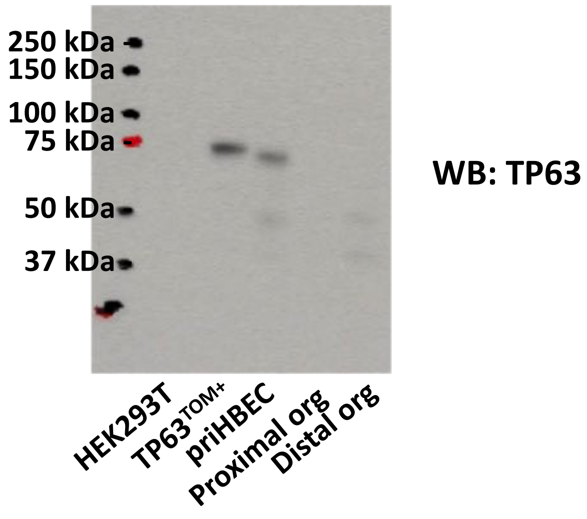

b

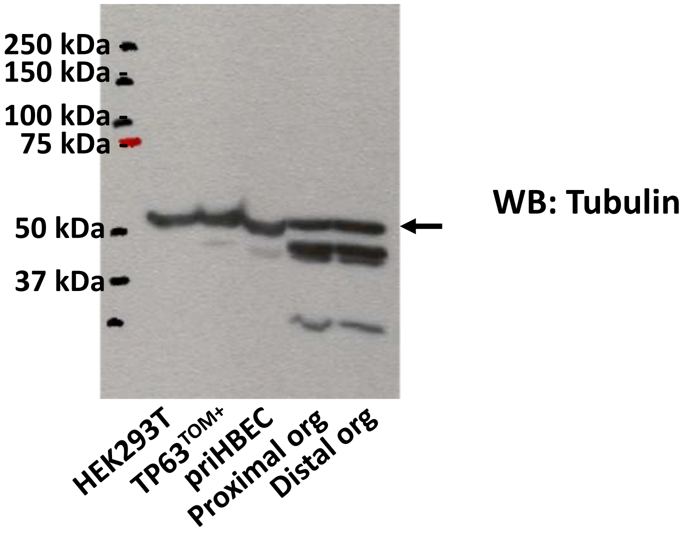

Supplementary Figure 5 - **Full blots of TP63, RFP (tdTomato) and Tubulin.**
30 µg of whole cell lysates prepared from HEK293T cells overexpressing tdTomato (HEK293T), $TP63^{TOM+}$ cells, primary human bronchial epithelial cells (priHBEC), proximalized (proximal org) and distalized lung organoids (distal org). Blots were probed with (a) anti-TP63 antibody and (b) anti-Tubulin antibody.

Supplementary figure 6

a

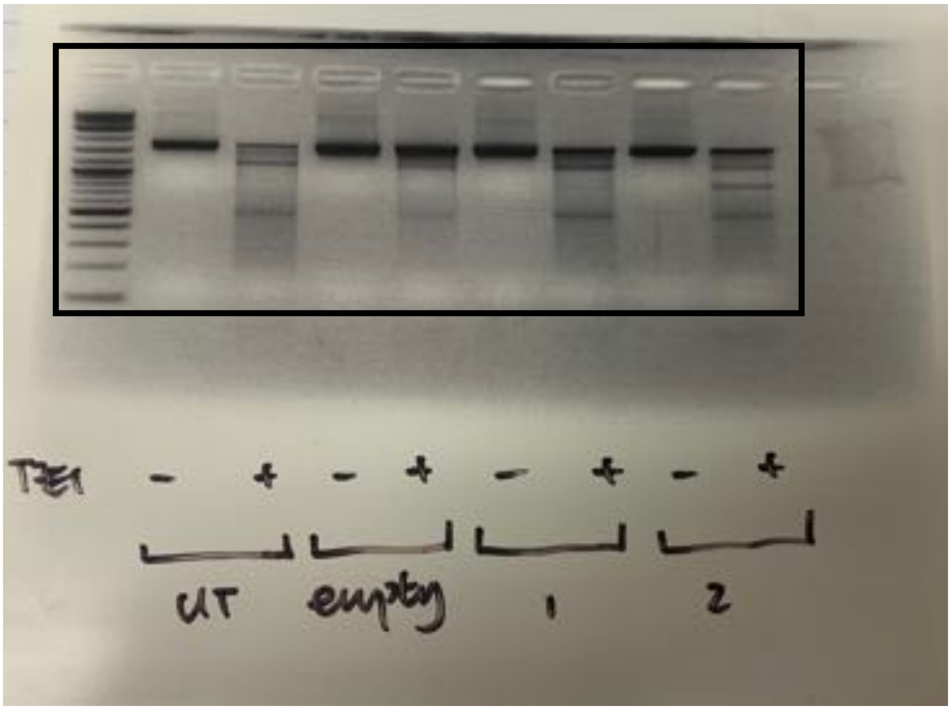

b

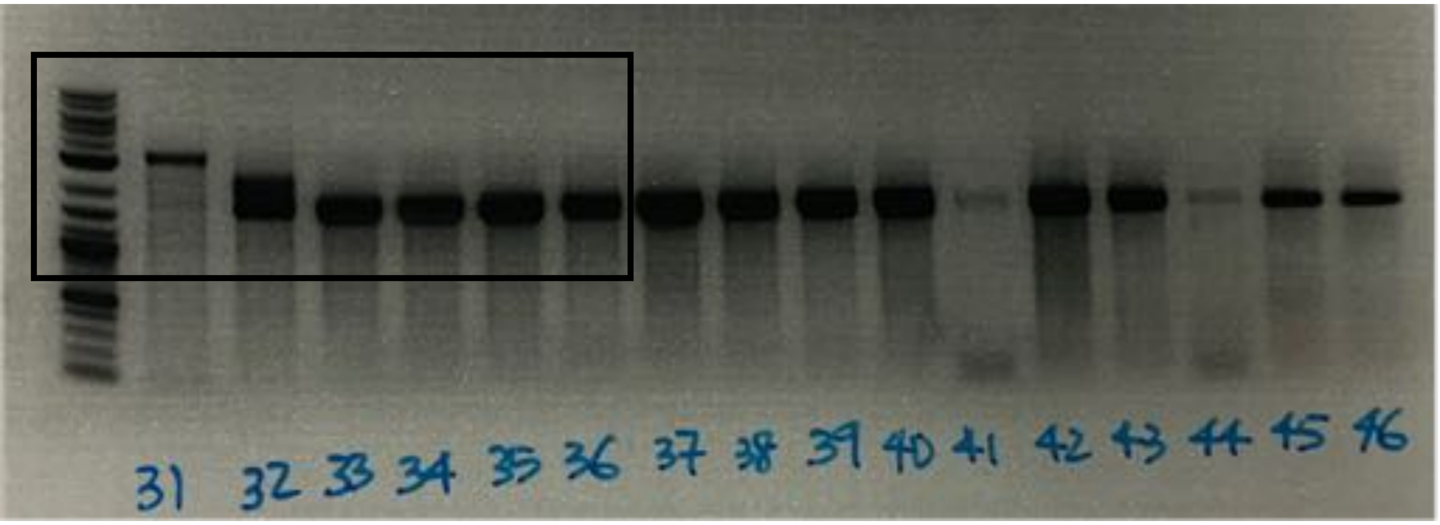

c

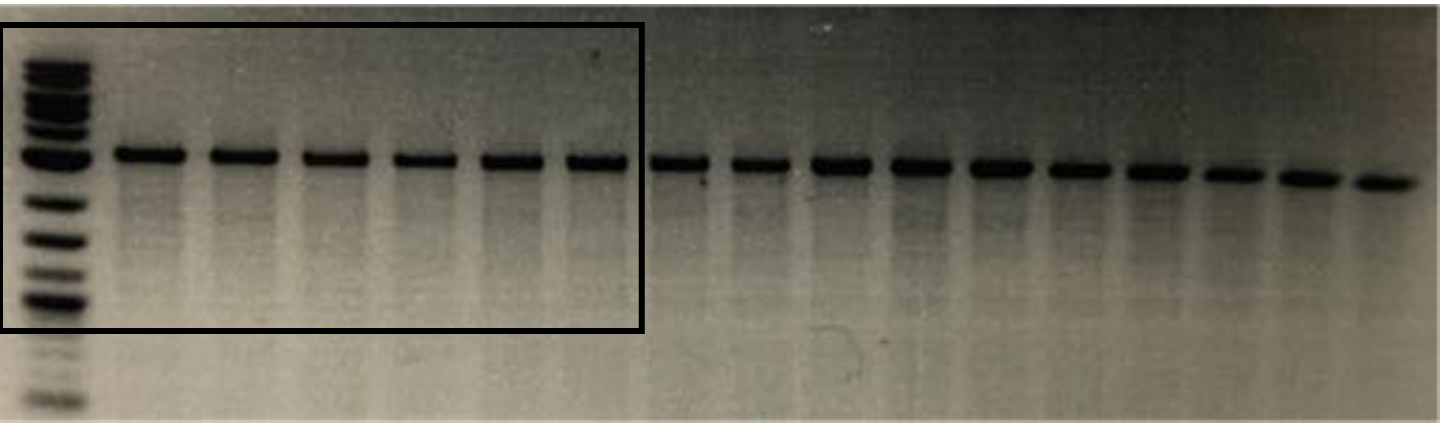

Supplementary Figure 6. Full gel images.
(a) Boxed region was presented in Supplementary Figure 1B.
(b) Boxed region was presented in Supplementary Figure 1C.
(c) Boxed region was presented in Supplementary Figure 1D.

Supplementary table 1 - List of PCR primer sequences

**sgRNA sequences**	
**Name**	**Sequence (5' → 3')**
sgRNA1_F	GCAGTGATGCGCTGTTGCTTATTG
sgRNA1_R	AAACCAATAAGCAACAGCGCATCA
sgRNA2_F	AAACCTTTGATGCGCTGTTGCTTA
sgRNA2_R	GCAGTAAGCAACAGCGCATCAAAG
**Primers used in the construction of donor template**	
**Name**	**Sequence (5' → 3')**
Ex14_tdTomato_STOP_UTR_F1	GTTAAAaCAAGCAGGAGATGTTGAAGAAAACCCtGGGCCcATGGTGAGCAAGGGCGAGGA
Ex1_tdTomato_STOP_UTR_F2	AGAGGAGGGGGAGGGATCcGGCGCCACaAACTTCagcCTGTTAAAaCAAGCAGGAGATG
tdTomato_SacI_R	TAGGAAGAGCTCACATGGTGAGGCTCACTT
tdTomato_Pre_SacI_R	GCTCACATGGTGAGGCTCACTTGTACAG
tdTomato_SacI_R2	GATAGGAAGAGCTCACATGGTGAGGCTCAC
Exon14_GSG_BamHI_F	GAGGAGGGGGAGGGATCcGGC
tdTomato_+718_F	ACCGCCTCCTCCGAGGACAACAA
sgRNA2_G791A_F	ACAGCGCATCAAAGAAGAGGGGGAGGGATC
sgRNA2_G791A_R	GATCCCTCCCCCTCTTCTTTGATGCGCTGT
**Primers used for genotyping and sequencing**	
**Name**	**Sequence (5' → 3')**
F primer	GTGACTTTAAACTTGTCAACCTCTCTGG
R primer	TATGCTCAAATACATGACGTCGGGTG
tdTomato_F	CTCCGAGGACAACAACATGG
tdTomato_R	CCATGTTGTTGTCCTCGGAG

Supplementary table 2 - List of antibodies

**Primary antibodies**					
**Target protein**	**Species**	**Application**	**Company**	**Catalogue No.**	**Dilution**
Acetylated tubulin	Mouse	IF	Sigma-Aldrich	T6793	1:100
AFP	Mouse	IF	Sigma-Aldrich	A8452	1:1000
MUC5AC	Mouse	IF	Abcam	ab3649	1:100
RFP	Rabbit	WB	Abcam	ab62341	1:1000
RFP	Rabbit	WB	Rockland	600-401-379	1:1000
SMA	Mouse	IF	Pierce	MA1-12772	1:1000
Tubulin	Mouse	WB	Sigma-Aldrich	T9026	1:5000
TUJ1	Mouse	IF	Covance	MMS-435P	1:1000
TP63	Rabbit	IF	Abcam	ab32353	1:100
TP63	Rabbit	WB	Abcam	ab124762	1:1000
**Secondary antibodies**					
**Target protein**	**Species**	**Application**	**Company**	**Catalogue No.**	**Dilution**
Alexa Fluor 647 Donkey anti-Mouse IgG (H+L)	Donkey	IF	Invitrogen	A31571	1:1000
Alexa Fluor 488 Donkey anti-Rabbit IgG (H+L)	Donkey	IF	Invitrogen	R37118	1:1000
Goat anti-rabbit HRP	Goat	WB	Dako	P044801	1:1000

Supplementary table 3 - List of qPCR primer sequences

Name	Forward Sequence (5' → 3')	Reverse sequence (5' → 3')
ACTB	CTGGAACGGTGAAGGTGACA	AAGGGACTTCCTGTAACAATGCA
AQP5	GCCATCCTTTACTTCTACCTGCTC	GCTCATACGTGCCTTTGATGATGG
CFTR	CTATGACCCGGATAACAAGGAGG	CAAAAATGGCTGGGTGTAGGA
EGFR	AGGCACGAGTAACAAGCTCAC	ATGAGGACATAACCAGCCACC
FOXJ1	CACGTGAAGCCTCCCTACTC	GGATTGAATTCTGCCAGGTG
F3	TCCCTCCCGAACAGTTAACC	CCCACTCCTGCCTTTCTACA
IL-33	GTGACGGTGTTGATGGTAAGAT	AGCTCCACAGAGTGTTCCTTG
ITGA6	TTTGAAGATGGGCCTTATGAA	CCCTGAGTCCAAAGAAAAACC
KRT5	GGAGTTGGACCAGTCAACATC	TGGAGTAGTAGCTTCCACTGC
MUC5AC	CCATTGCTATTATGCCCTGTGT	TGGTGGACGGACAGTCACT
NGFR	CCTGGACAGCGTGACGTTC	CCCAGTCGTCTCATCCTGGT
NKX2-1	ACCAGGACACCATGAGGAAC	CGCCGACAGGTACTTCTGTT
PDPN	GTCCACGCGCAAGAACAAAG	GGTCACTGTTGACAAACCATCT
SCGB3A2	CAAGTGGAACCACTGGCTTG	CCAGAGGTAAAGGTGCCAAC
SFTPC	CACCTGAAACGCCTTCTTATCG	TGGCTCATGTGGAGACCCAT
SYP	TTTGTGAAGGTGCTGCAATG	ACCTCGATGCTGAGGTCACT
S100A2	GCGACAAGTTCAAGCTGAGT	ACAGTGATGAGTGCCAGGAA
TP63 (total)	AAAGACATGCCCCATCCAGA	CATACTGGGCATGGCTGTTC
TP63α	TGCAGCATTGTCAGTTTCTTAGC	TGCTCAATCTGATAGATGGTGGT
TP63β	TTGCAGCATTGTCAGGATCTGG	AGAAGGGGAGGAGAATTCGT
TP63γ	GGAACTCATGCAGTACCTTCCT	TTCCTGAAGCAGGCTGAAAGG
ΔNP63	ATTCATATTGTAAGGGTCTCGGG	GGGCATTGTTTTCCAGGTACAA
TAP63	AATTTTGAAACTTCACGGTGTGC	TCTTTCCAAGAGAAATGAGCTGG
tdTomato	CCACAAACTTCAGCCTGTTAAA	GCGCATGAACTCTTTGATGAC

Supplementary video

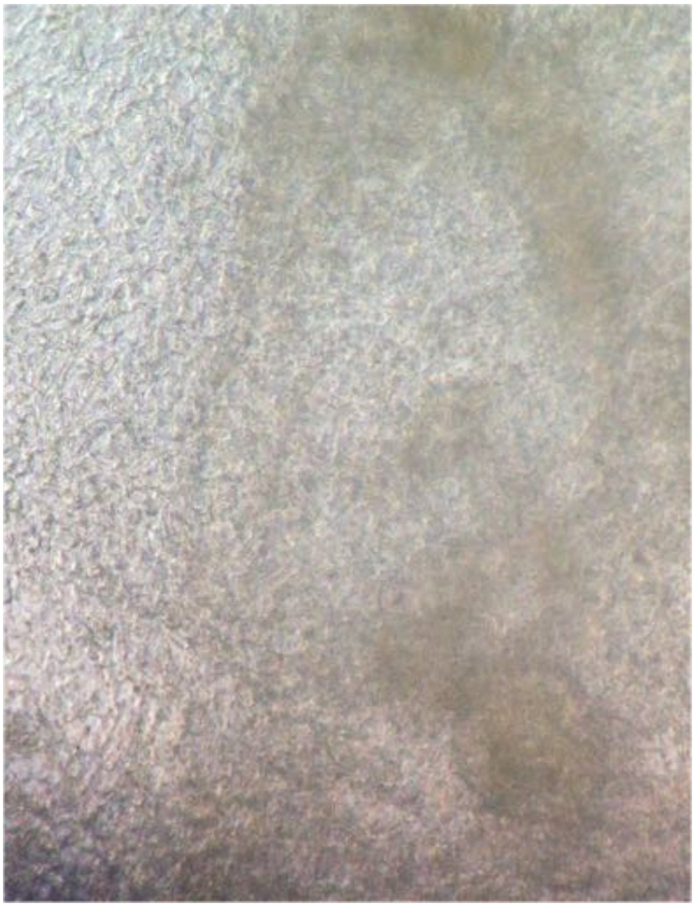

Supplementary video.
Ciliary movement observed in DP cells differentiated in transwells at ALI at Day 45, captured using Leica DMi1 microscope at 100X magnification.
